# Cost-Effectiveness of HBV and HCV Screening Strategies – A Systematic Review of Existing Modelling Techniques

**DOI:** 10.1371/journal.pone.0145022

**Published:** 2015-12-21

**Authors:** Claudia Geue, Olivia Wu, Yiqiao Xin, Robert Heggie, Sharon Hutchinson, Natasha K. Martin, Elisabeth Fenwick, David Goldberg, Consortium and ECDC

**Affiliations:** 1 Health Economics and Health Technology Assessment, Institute of Health and Wellbeing, University of Glasgow, Glasgow, United Kingdom; 2 School of Health and Life Sciences, Glasgow Caledonian University, Glasgow, United Kingdom; 3 Division of Global Public Health, University of California San Diego, San Diego, California, United States of America; 4 School of Social and Community Medicine, University of Bristol, Bristol, United Kingdom; 5 ICON Health Economics and Epidemiology, Dublin, Ireland; 6 Health Protection Scotland, NHS Health Scotland, Glasgow, United Kingdom; The Chinese University of Hong Kong, HONG KONG

## Abstract

**Introduction:**

Studies evaluating the cost-effectiveness of screening for Hepatitis B Virus (HBV) and Hepatitis C Virus (HCV) are generally heterogeneous in terms of risk groups, settings, screening intervention, outcomes and the economic modelling framework. It is therefore difficult to compare cost-effectiveness results between studies. This systematic review aims to summarise and critically assess existing economic models for HBV and HCV in order to identify the main methodological differences in modelling approaches.

**Methods:**

A structured search strategy was developed and a systematic review carried out. A critical assessment of the decision-analytic models was carried out according to the guidelines and framework developed for assessment of decision-analytic models in Health Technology Assessment of health care interventions.

**Results:**

The overall approach to analysing the cost-effectiveness of screening strategies was found to be broadly consistent for HBV and HCV. However, modelling parameters and related structure differed between models, producing different results. More recent publications performed better against a performance matrix, evaluating model components and methodology.

**Conclusion:**

When assessing screening strategies for HBV and HCV infection, the focus should be on more recent studies, which applied the latest treatment regimes, test methods and had better and more complete data on which to base their models. In addition to parameter selection and associated assumptions, careful consideration of dynamic versus static modelling is recommended. Future research may want to focus on these methodological issues. In addition, the ability to evaluate screening strategies for multiple infectious diseases, (HCV and HIV at the same time) might prove important for decision makers.

## Introduction

The effectiveness and cost-effectiveness of screening for infectious diseases such as Hepatitis B (HBV) and Hepatitis C (HCV) have been evaluated in a number of studies [1–3]. However, studies are generally heterogeneous, in terms of the populations studied (e.g. different risk groups in different countries), the screening strategies adopted (different means of testing in a variety of clinical and community settings) and the outcomes measured (e.g. infections detected, life-years gained and quality adjusted life-years (QALYs) gained). Studies further differ in terms of the methods employed in order to evaluate the cost-effectiveness of screening. Cost-effectiveness results may depend on the type of economic model used and also on assumptions made regarding model structure and input parameters. The aims of this study were to undertake a systematic review, summarise and critically assess the existing economic models for HBV and HCV in order to identify the main methodological differences in modelling approaches.

## Methods

A systematic review was carried out according to the principles set out by the Campbell and Cochrane Economics Methods Group (CCEMG) [4].

### Eligibility Criteria

Studies fulfilling the following criteria were included in the systematic review:

· Population—the general population (excluding blood donors) and selected subpopulations (women during pregnancy, men who have sex with men (MSM), immigrants, injecting drug users (IDUs), recipients of blood transfusion and blood products and healthcare workers (HCW)) from OECD countries.
· Intervention–testing strategies for HBV or HCV in different settings.
· Comparator–no testing or alternative testing strategies.
· Outcomes–measurement and reporting of both costs and benefits (regardless of how they were measured).
· Study type–economic evaluation incorporating cost-effectiveness analysis.

No language restrictions were imposed during the literature search.

A structured search strategy was developed under the guidance of a specialist subject librarian (S1 Text) and relevant search filters that are highly sensitive and tailored to specific databases were incorporated [5]. Separate search strategies were developed for HBV and HCV screening. A multi-facetted approach for identifying the relevant literature was carried out; the following data sources were searched from inception to November 2011 and updated to July 2015: MEDLINE, EMBASE, Cumulative Index to Nursing and Allied Health Literature (CINHAL), Health Technology Assessment (HTA) and Economic Evaluation Database (NHS EED), Database of Abstracts of Reviews of Effects (DARE), European Network of Health Economics Evaluation Databases (EURONHEED). This was supplemented by using the Web of Science database to generate a list of articles that cited identified original studies. Hand searching the references of all studies meeting the inclusion criteria was also carried out.

### Data Extraction/Collection process

Screening of titles and abstracts was carried out, followed by selecting relevant studies based on *a priori* identified inclusion and exclusion criteria. Two independent reviewers selected and reviewed all relevant studies and assessed their quality. Study results were systematically extracted by one reviewer according to a pre-defined protocol and summarised in evidence tables. Data extraction was subsequently validated independently by a second reviewer. Any disagreement relating to the inclusion of studies, data extraction, or quality assessment between the reviewers was resolved by discussion.

### Model Critique

A critical assessment of the decision-analytic models was carried out according to the guidelines and framework developed for assessment of decision-analytic models in Health Technology Assessment of health care interventions [6]. The checklist developed by Philips and colleagues provides a structure for any critical appraisal of economic models and concentrates on model structure. This was used in addition to the Drummond Checklist [6, 7]. A performance matrix was developed based on the checklist developed by Philips et al. The matrix summarises various elements from the checklist into twelve distinct categories in order to reflect the overall quality of the model (S2 Text).

## Results

### Cost-effectiveness analyses of screening for HBV

**Fig 1:**
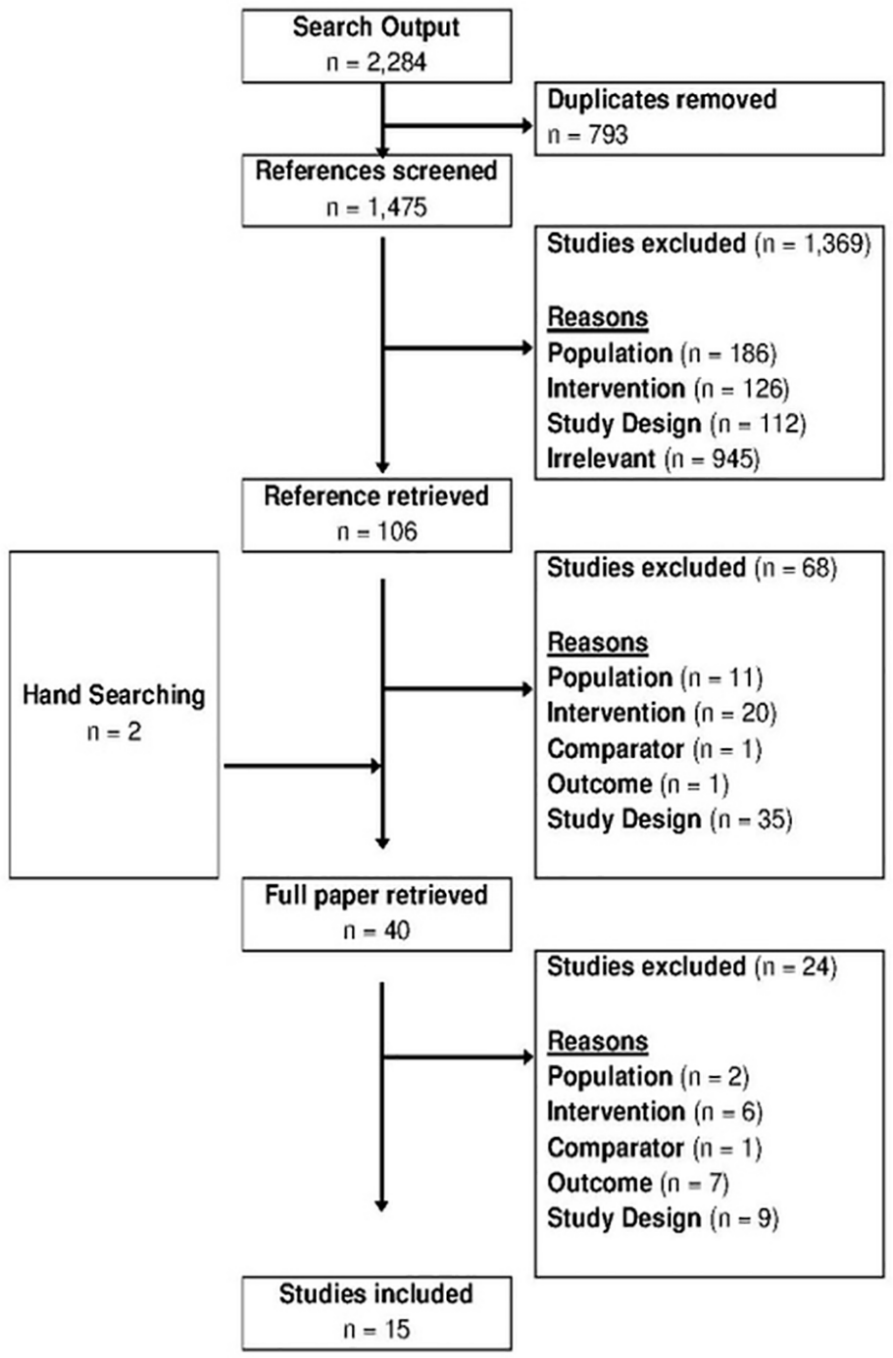
Selection of studies on cost-effectiveness of HBV screening until November 2011.

**Fig 2:**
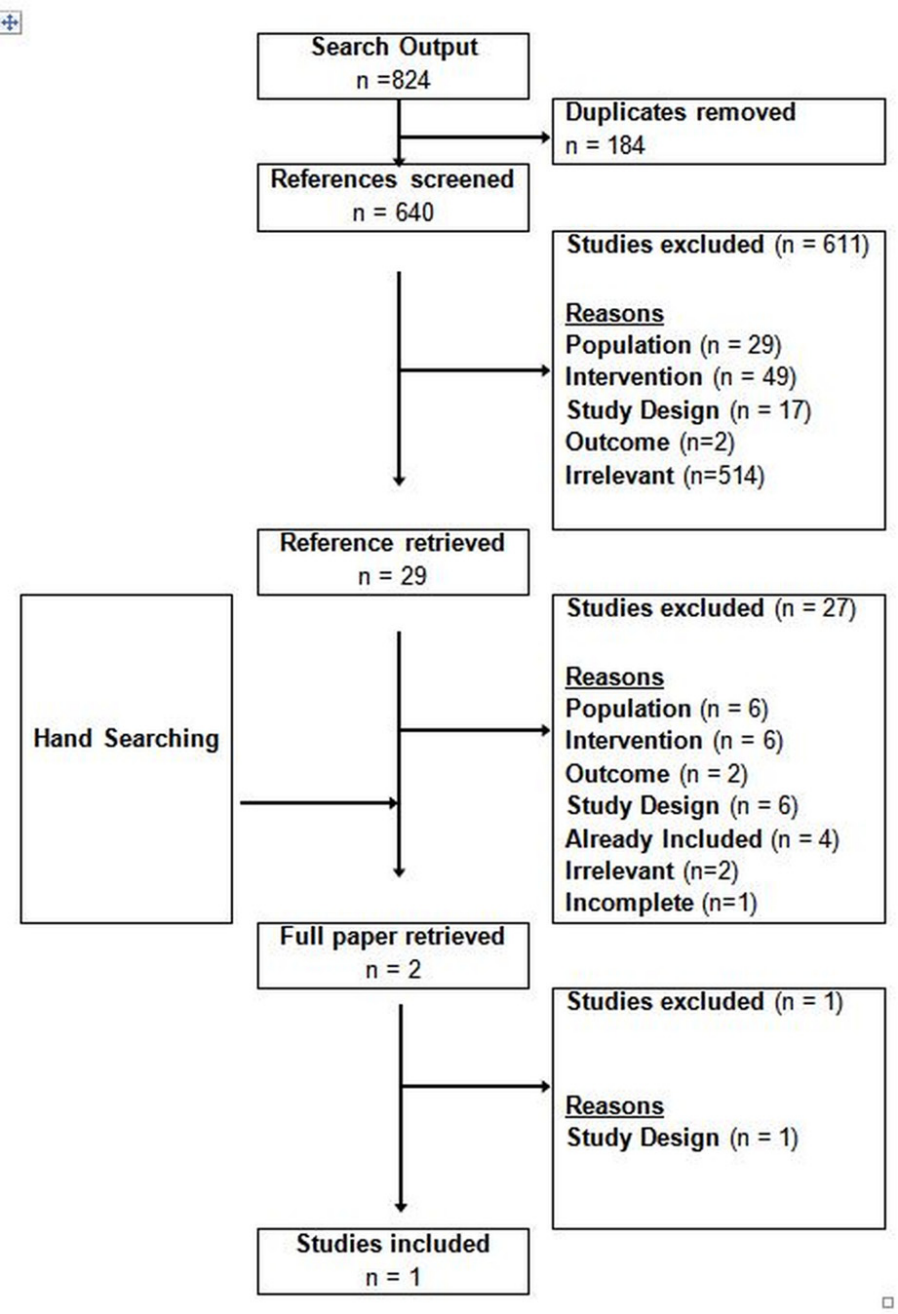
Selection of studies on cost-effectiveness of HBV screening; September 2011 until July 2015.

The initial search (up to November 2011) returned 2,284 references, of which 15 studies met the inclusion criteria (Fig 1). The literature search was subsequently updated in July 2015 and returned 824 references. One additional study was identified (Fig 2). Studies evaluated the cost-effectiveness of screening for HBV in Europe, North America and Australia in different settings and populations. Five studies evaluated screening in the general population [8–12], three studies looked at women during pregnancy [13–15], five studies analysed screening for immigrants [16–20] and another three studies [21–23] looked at screening newborns, preadolescents and adolescents. Studies predominantly assessed screening strategies in combination with vaccination and treatment.

All cost-effectiveness results have been summarised and are presented in Table 1 below. Detailed data extraction tables are available as S1 Table.

**Table 1:** Summary of Cost-Effectiveness Results- HBV.

Study	Strategies Compared		Prevalence	Costs for Strategies		Outcomes for Strategies		ICER
Antonanzas, 1995	Mass immunisation of adolescents	Do nothing	Varied by age	N/A		N/A		N/A
	Mass immunisation of infants	Do Nothing	Varied by age	N/A		N/A		N/A
	Combined mass immunisation	Do Nothing	Varied by age	N/A		N/A		N/A
	Screening all women during pregnancy combined with above strategies	Do Nothing	Varied by age	N/A		N/A		N/A
Arevalo, 1988	Screening & vaccination (at birth and repeated hepatitis vaccine at months 1 and 6)	Do Nothing	0.2%	$1,157,000	$7,950,000	Cases prevented2-140	0	N/A
Bloom, 1993	Screening & Vaccination High risk newborns and all adolescents	Do Nothing		Not provided		27.8	0	$3,695 per LYG
	Screening & Vaccination General adult population	Do Nothing		Not provided		4.8	0	$279,184 per LYG
	Screening & Vaccination High risk adults	Do Nothing		Not provided		32.6	0	Cost saving
	Screening & Vaccination Newborns	Do Nothing		Not provided		18.5	0	$42,067 per LYG
	Vaccination all adolescents	Do Nothing		Not provided		13.9	0	$97,256 per LYG
	Vaccination General adult population	Do Nothing		Not provided		5.8	0	$257,418 per LYG
	Vaccination High risk adults	Do Nothing		Not provided		52.5	0	Cost saving
	Vaccination Newborns	Do Nothing		Not provided		17	0	$38,632 per LYG
Eckman, 2011	Screen and treat with low cost nucleoside/ nucleotide (general population)	No screening	0.4%	$1,177.963	$914.76	23.2319	23.2228	$29,232 per QALY gained
Hutton, 2007	Screen, treat and ring vaccinate, Immigrants (Asian and Pacific Islanders)	Screen and treat	10%	$868,612,000	$866,204,000	237,909	237,849	$39,903 per QALY gained
	Screen and treat Immigrants (Asian and Pacific Islanders)	Status quo	10%	$866,204,000	$846,008,000	237,849	237,289	$36,088 per QALY gained
Kim, 2006	Routine Vaccination (CTSs) -(General population of high risk adults)	No Intervention	Not specified	$680,000	$0	155	0	$4,400 per QALY gained
	Screening & Vaccination	Routine Vaccination		$850,000	$680,000	61	155	Dominated
	Screening with initial dose	Routine vaccination		$680,000	$1,220,000	152	155	Dominated
	Routine Vaccination (STD clinic)—(General population of high risk adults)	No Intervention		$740,000	$0	214	0	$3,500 per QALY gained
	Screening & Vaccination	Routine Vaccination		$960,000	$740,000	107	214	Dominated
	Screening with initial dose	Routine vaccination		$1,320,000	$740,000	209	214	Dominated
Kwan-Gett, 1994	Pre-vaccination testing	N/A	Not specified	N/A		N/A		N/A
	Test & Vaccinate	N/A		N/A		N/A		N/A
	No Testing	N/A		N/A		N/A		N/A
Mulley, 1982	No Intervention (MSM)	n/a	5%	N/A		N/A		N/A
Rein, 2011	Model 1	N/A	1.7% /6.3%	Cost per case identified: $609		N/A		N/A
	Model 2	N/A	1.7% /6.3%	Cost per case identified: $1,584		N/A		N/A
	Model 3	N/A	1.7% /6.3%	Cost per case identified: $3,150		N/A		N/A
	Model 4	N/A	1.7% /6.3%	Cost per case identified: $4,657		N/A		N/A
Ruggeri, 2011	Test (high risk population)	No Test	7%	€67,007.73	€7,939.39	20.07	16.63	€18,255.97 per QALY gained
Thomas, 1990	Universal screening during pregnancy	N/A		Per carrier identified:$354		N/A		N/A
	Screening of high risk women during pregnancy	N/A		Per carrier identified:$97		N/A		N/A
	Screening of low risk women during pregnancy only	N/A		Per carrier identified:$2,005		N/A		N/A
Tormans, 1993	Screening & vaccination during pregnancy	Do nothing	0.67%	BEF31,719,490	BEF 6,800,587	5.93 (LYL)	48.63 (LYL)	BEF 583,581 per LYG
Veldhuijzen, 2010	One-off systematic screening and treatment of eligible patients (immigrants)	Status quo of no screening	3.35%	€168,480,000	€109,178,000	120,025	113,411	€8,966 per QALY gained
Wong, 2006	Screen & Treat (Tenofovir)	No screening	4.81%	$(CAD)74,911	$ (CAD)73,246	16.17	16.15	$ (CAD)69,209 per QALY gained
	Screen, treat & vaccinate (Tenofovir)	Screen & Treat	4.81%	$(CAD)74,992	$(CAD)74,911	16.17000022	16.17	$(CAD)3,648,123 per QALY gained
	Screen & Treat (Entecavir)	No screening	4.81%	$(CAD)75,380	$(CAD)73,352	16.17	16.15	$(CAD)101,513 per QALY gained
	Screen, treat & vaccinate (Entecavir)	Screen & Treat	4.81%	$(CAD)75,447	$(CAD)75,380	16.1700028	16.17	$(CAD)241,983 per QALY gained
Zurn, 2000	Universal school children vaccination	Systematic screening & selective vaccination	5%	Not specified		Not specified		SwF10,200 per LYG
	Universal vaccination of infants	Systematic screening & selective vaccination	5%	Not specified		Not specified		SwF6,120 per LYG
	Universal school children vaccination	Universal vaccination of infants	5%	Not specified		Not specified		SwF10,480 per LYG
	Universal vaccination of adolescents	Universal vaccination of infants & school children	5%	Not specified		Not specified		SwF10,480 per LYG
Rossi, 2013	Universal vaccination	Do nothing	6.5%	$5,472	$5,429	21.7462 QALYs	21.7463 QALYs	Dominated
	Screening for prior immunity and vaccination	Do nothing	6.5%	$5,485	$5,429	21.7462 QALYs	21.7463 QALYs	Dominated
	Chronic HBV screening and treatment	Do nothing	6.5%	$6,077	$5,429	23.6292 QALYs	21.7463 QALYs	$40,880 per QALY
	Combined screening for chronic HBV and prior immunity, treatment and vaccination	Chronic HBV screening and treatment	6.5%	$6,101	$6,077	23.6293 QALYs	23.6292 QALYs	$437 per QALY

Studies carried out in the general population mainly evaluated the cost-effectiveness of screening versus no screening and the cost-effectiveness of different vaccination strategies. All identified economic evaluations were based on decision-analytic models consisting of decision trees and/or Markov models. The decision tree usually reflected the screening strategy and outcomes were presented as ‘cases identified’ while the Markov model added the disease and treatment elements with outcomes usually reflected as QALYs. The measure of health benefits varied across studies, and cost-effectiveness was expressed as for instance: incremental costs per case averted [11], life-years gained [8, 15, 23] or QALY gained [9, 10, 12, 16, 18, 19]. Compared with no screening, screening for HBV in high-risk groups (prevalence >5%) of the general population has been shown to be cost-effective in an Italian and a US study [10, 12].

Two studies (US and Belgium) used decision trees to show that screening women during pregnancy and subsequent immunisation of infants of test-positive mothers was cost-effective compared to no screening [13, 15]. The Belgian study was carried out from a healthcare system perspective and cost-effectiveness was expressed as incremental costs per life-year saved [15]. The US study provided a cost and cost-effectiveness was expressed as total net costs; health outcomes were expressed as total net costs with and without screening and vaccination, taking into account potential adverse outcomes prevented [13]. This study reported that universal screening of women during pregnancy and subsequent immunisation of infants compared with no screening or vaccination was considered cost-saving in a US context (over $3 million (US dollars) saved per 100,000 women screened).

In another study, universal screening and vaccination was also compared with selective screening of high-risk pregnant women [14]. In the absence of a decision-analytic model or an incremental analysis, this study carried out naïve comparisons between the costs per carrier identified with universal and that with selective screening, and vaccination in women during pregnancy in one antenatal clinic (N = 5,858). Thomas et al estimated that $18,394 (Australian dollars; AUD) would be required to detect 52 positive cases.

Five studies assessed the cost-effectiveness of screening for HBV among migrants, in a hypothetical immigration cohort in Canada [19, 20]; in migrants from countries with high and intermediate HBV prevalence compared with the status quo in the Netherlands [18]; in immigrants from countries with high HBV prevalence in the US [17]; and in Asian and Pacific Islanders living in the US [16]. In particular, Hutton et al took into account potential vertical transmissions of mother to infants [16]. In four studies, incremental cost-effectiveness analyses were carried out, and the incremental costs per QALY gained between strategies were estimated [16, 18–20]. Rein et al based their analysis on six months of observational data. The cost per person screened was calculated, but no formal cost-effectiveness analysis was undertaken [17]. Screening and treating Asian and Pacific Islanders was shown to be a cost-effective strategy compared with the status quo [16]. Incorporating screening and treatment of close contact individuals of those tested positive also showed cost-effective results (ICER of $39,903 per QALY gained) [16]. Similarly, screening adult migrants soon after arrival in Canada for chronic HBV and providing antiviral treatment was shown to be cost-effective with an ICER of 40,880 Canadian dollars per QALY gained [20]. The remaining studies showed similar favourable results.

Various screening strategies were evaluated in newborns, preadolescents and adolescents including: pre-vaccination testing [22], primary prevention and screening [21], vaccinating high risk groups [23]. These were compared to an array of comparators, such as: ‘do nothing’, prenatal screening and subsequent vaccination of newborns at risk, universal vaccination of infants, school children and adolescents [21–23]. These studies either used a decision tree [22, 23] and compartmental and Markov models [21]. Conclusions on cost-effectiveness were mixed and difficult to generalise as studies used a range of outcome measures, making it difficult to compare between studies.

### Critical appraisal of economic models–Hepatitis B Virus

**Fig 3:**
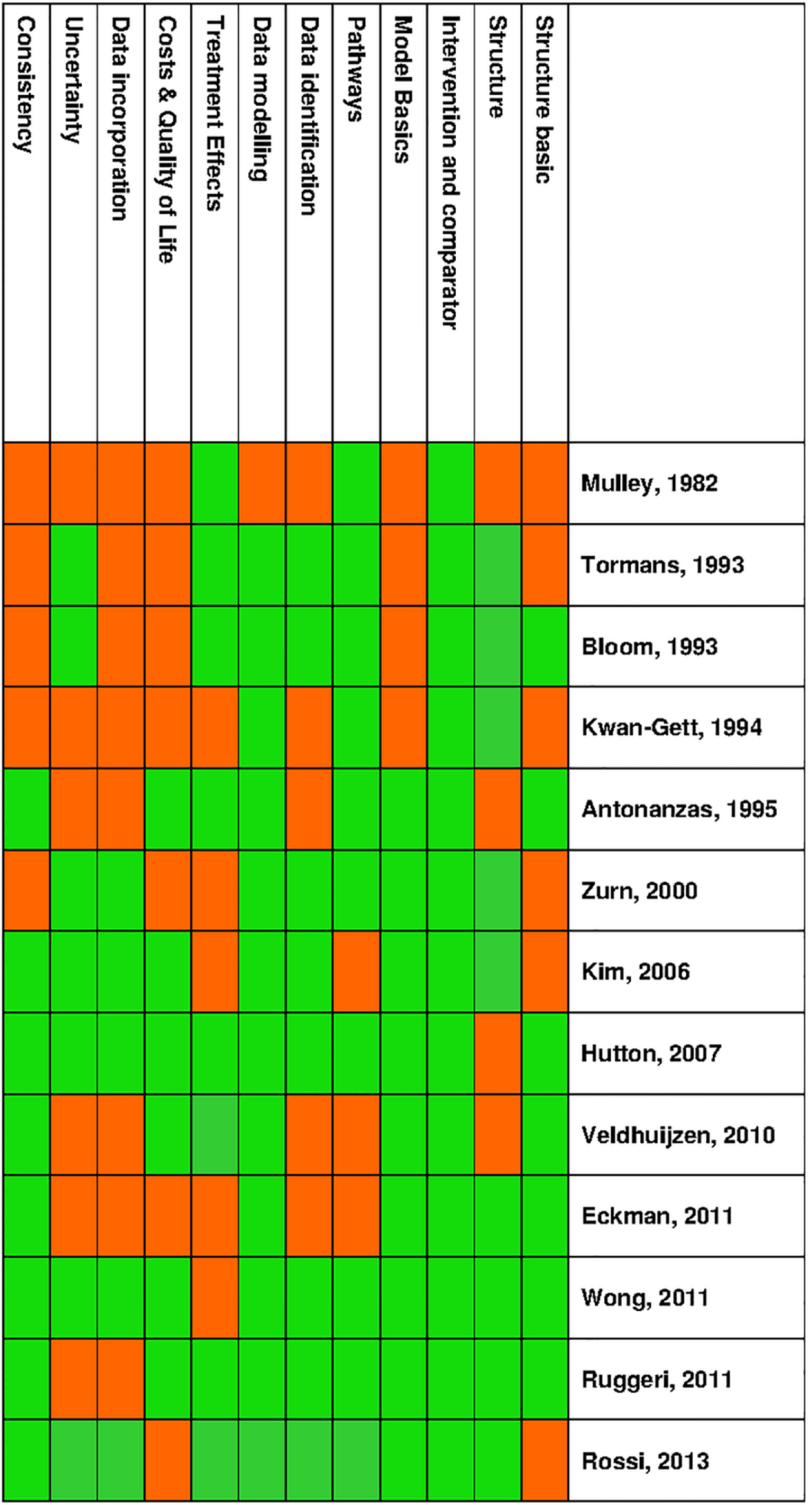
Performance Matrix—HBV screening models.

The checklist developed by Philips et al was completed for those studies that used an economic model to estimate cost effectiveness of screening (n = 13). The associated performance matrix (Fig 3) shows results for each individual study and their performance in each of the twelve distinct categories of the checklist. Overall, the quality of the models varied greatly. There are however common trends, with more recent studies performing better than earlier studies. Model inputs were generally consistent with the stated perspective which included that of the payer (taking into account direct medical costs) or that of the society (taking into account direct medical costs and costs to the individuals being screened or vaccinated). Although the costs for carrying out screening of patients were included in the analyses, most studies neglected the costs associated with various screening efforts (i.e. costs associated with efforts of recruiting patients).

Data sources used to develop the structure of the model were usually specified, but studies rarely reported whether the quality of the data used in the models had been assessed. Studies mainly sourced their data from the literature but also used expert opinion [21, 23]. Studies generally provided a graphical representation of a model, which showed the transition of individuals between health states or the key alternatives of the decision (screening strategy) and its associated consequences. A number of studies either did not report their time horizon or used a time horizon which was judged not to be sufficient to reflect all important differences between the options under evaluation [14, 15, 21, 22]. Further variations that were not accounted for by study location were found in the discount rates used for future costs and benefits.

Results for any cost-effectiveness analyses depend on assumptions made during the modelling process and key assumptions are summarised below.

Prevalence estimates are difficult to obtain as the underlying prevalence in a population is unknown and studies frequently relied on estimates obtained from national surveys or the published literature. With prevalence being the main driver of cost-effectiveness results, interpretation always needs to be made in relation with the prevalence rate employed in the model. Sensitivity of the model results to the level of prevalence, was not always tested.

Assumptions made on the protection rate of a vaccine and assumptions made on the proportion of individuals protected from HBV by virtue of a previous infection are important. Limitations were identified in one study’s ability to capture the full benefits and limitations of vaccination such as the indirect effects of herd immunity [19]. The intensity of the infection, which was assumed to be constant, and the annual probability of acquiring HBV infection, which was also assumed to be constant, were both identified as important parameters in another study [16]. With both, the assumption of a constant rate was found to limit the study’s ability to infer precise cost-effectiveness results [16].

In addition, compliance rates together with the length of efficacy of the vaccine (e.g. 10 years) and efficacy of the treatment were found to impact on the disease part of the economic model [20]. It was further assumed that vaccines do not incur any side effects that required medical care, thus impacting in particular on the treatment costs. Studies have made simplifying assumptions on test sensitivity and specificity [8]. Although standards should apply for these values, variations might be found depending on whether tests are carried out correctly. This would impact on the proportion of patients correctly identified with the virus and referred for treatment. Further limitations extend to the fact that death unrelated to HBV was not always included.

### Cost-effectiveness analyses of screening for HCV

**Fig 4:**
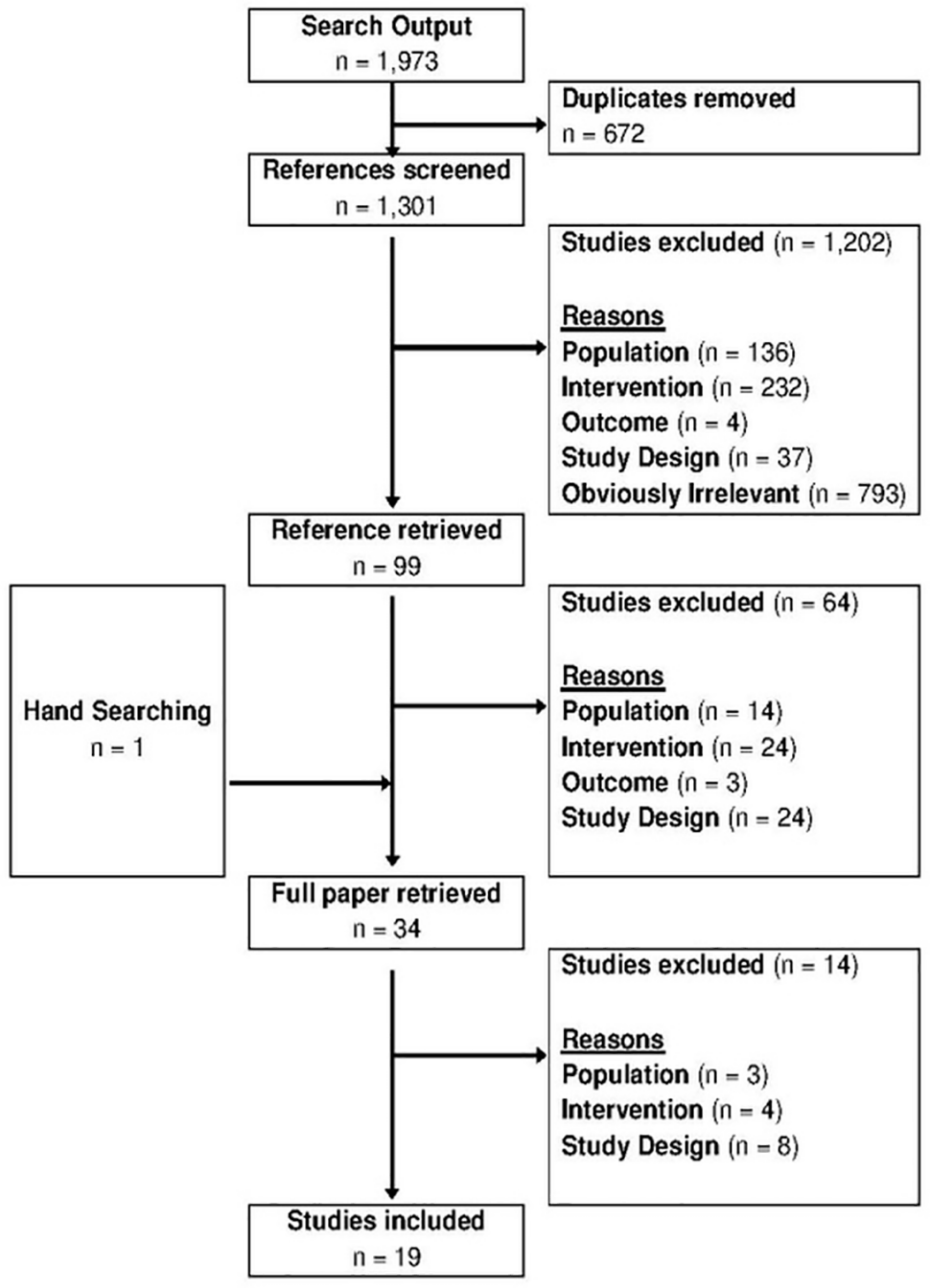
Selection of studies on cost-effectiveness of HCV screening until November 2011.

**Fig 5:**
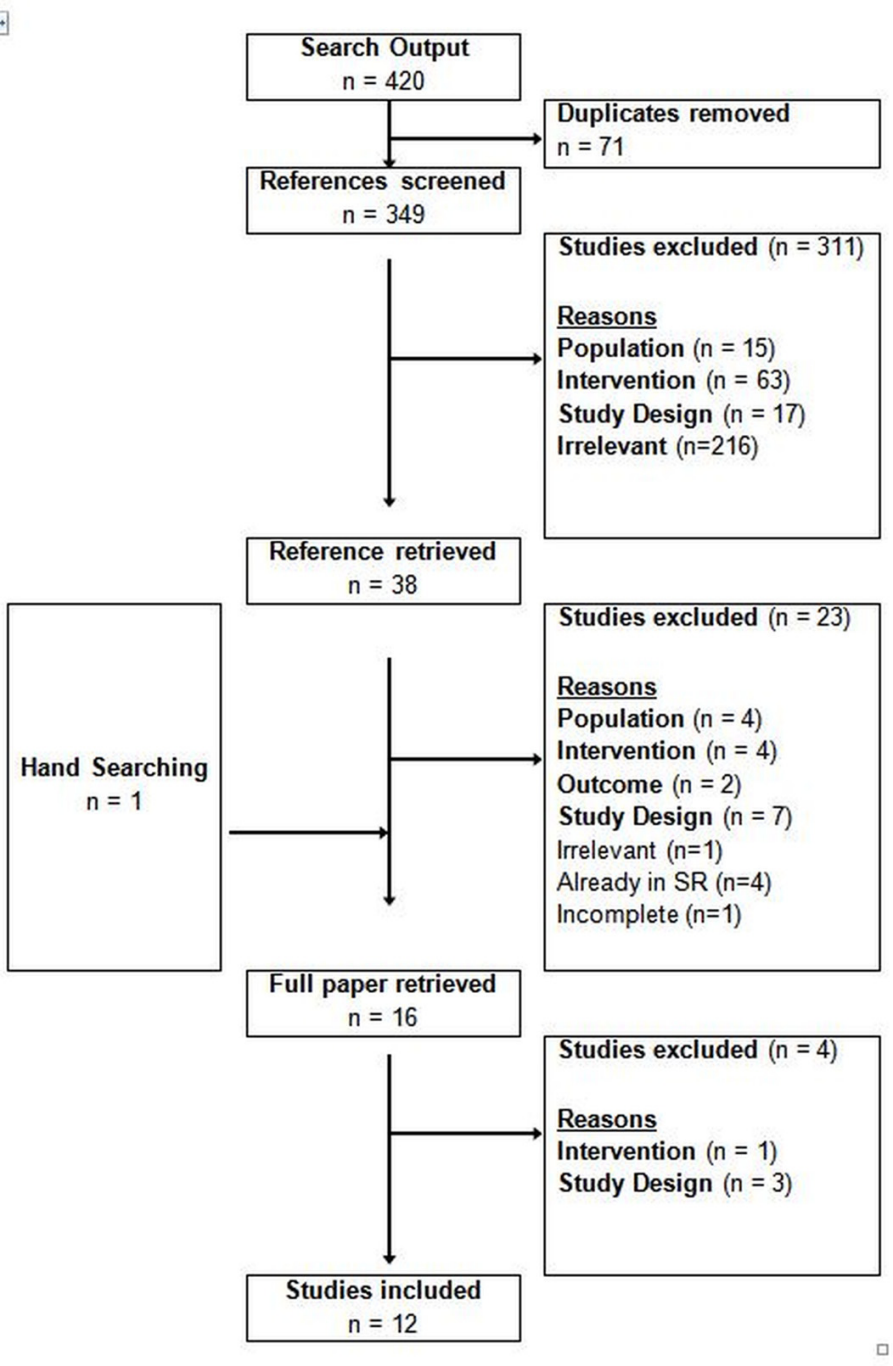
Selection of studies on cost-effectiveness of HCV screening; September 2011 until July 2015.

The search (up until November 2011) identified 1,973 references of which 19 studies met the inclusion criteria and were included in the review (Fig 4). The updated search in July 2015 returned a total of 420 references of which 12 studies were added to the review (Fig 5). The majority of studies evaluated the cost-effectiveness of screening for HCV in Europe or the US; one study was carried out in Japan. Fourteen studies evaluated screening in the general population [24–37]; 13 studies investigated screening IDUs [24, 26, 27, 38–47]; three studies looked at recipients of blood transfusions [26, 27, 48]; two studies evaluated screening in women during pregnancy [49, 50], while one study included first-generation pregnant migrants from non-Western countries [50], one study evaluated cost-effectiveness of screening migrants [51], one looked at HIV positive MSM [52] and a further study looked at HCWs [53]. It has to be noted here, that a number of studies evaluated the cost-effectiveness of HCV screening in multiple population groups (i.e. 22 studies reported on 26 cost-effectiveness analyses in five population groups).

Results of all cost-effectiveness analyses have been summarised and are shown in Table 2. Detailed results for data extraction can be found as S2 Table.

**Table 2:** Summary of Cost-Effectiveness Results–HCV.

Study	Strategies Compared		Prevalence	Costs for Strategies		Outcome for Strategies		ICER
Castelnuovo, 2006	Testing for HCV infection and offer antibody and if necessary RNA testing (former IDUs)	No screening	Male: 54% Female: 51% All: 53%	£2,443,336	£1,613,513	9,119	9,071	£17,515 per QALY gained
	Testing for HCV infection and offer antibody and if necessary RNA testing (Prisoners)	No screening	Male: 36%Female: 33%All: 35%	£769,912	£515,165	2,906	2,892	£20,083 per QALY gained
Coffin et al, 2012	Universal screening(15%)	Risk -based screening	0.5%	$60,269	$59,938	13.54	13.50	$ (US)7,900 per QALY gained
	Universal screening(60%)	Risk -based screening	0.5%	$61,437	$59,938	13.63	13.50	$ (US)10,900 per QALY gained
Deuffic-Burban, 2009	French recommendation (HCWs)	Alternative US recommendation (HCWs)	Not specified	€181.40	€178.50	23.2396	23.2549	Dominated
	European recommendation(HCWs)	Alternative US recommendation (HCWs)	Not specified	€155.10	€178.50	23.2409	23.2549	Dominated
	Alternative US (HCWs)	Baseline US recommendation(HCWs)	Not specified	€178.50	€126.60	23.2549	23.2292	€2,020 per QALY gained
Honeycutt, 2007	HCV counselling, testing and referral (IDUs)	n/a	57%	N/A		N/A		N/A
	HCV counselling, testing and referral (Men ≥40 years with history of ≥100 sexual partners)	n/a	16%	N/A		N/A		N/A
	HCV counselling, testing and referral (Men ≥40 years with <100 lifetime sexual partners)	n/a	2%	N/A		N/A		N/A
	HCV counselling, testing and referral (Women ≥40 years)	n/a	0.9%	N/A		N/A		N/A
Josset, 2004	**PA96 (**medical practices observed before 1996 in France (Blood Transfusion Recipients)	**C3** (screening before transfusion and three months after transfusion)	Not specified	€21,571	€71,898	56	137	€619 per case identified
	**PA96 (**medical practices observed before 1996 in France (Blood Transfusion Recipients)	**C6** (screening before transfusion and six months after transfusion)		€21,571	€67,047	56	137	€559 per case identified
	**PA96 (**medical practices observed before 1996 in France (Blood Transfusion Recipients)	**Cpt** (screening before transfusion only)		€21,571	€48,405	56	134	€343 per case identified
	**Cpt** (screening before transfusion only)	**C6** (screening before transfusion and six months after transfusion)		€48,405	€67,047	134	137	€5,825 per case identified
Josset, 2004	Screening in various risk groups	2.6%	Mean cost per case of HCV infection detected				N/A
Jusot, 2001	Screening via alanine aminotransferase (ALT), 3^rd^ generation enzyme immuneassay (EIA 3) or detection of HCV RNA in various groups	N/A	3%	N/A		N/A		N/A
Lapane et al, 1998	Test offered to those who had >7% probability of having HCV, according to a mathematical predictive equation.	Test offered to those at significant risk, according to responses to all the questions, with the exception of socially intrusive questions, of a survey.	20%	4.4	3.5	$1,571	$1,706	Dominates
	Test offered to those at significant risk, according to responses to all the questions of a survey.	Test offered to those at significant risk, according to responses to all the questions, with the exception of socially intrusive questions, of a survey.	29%	4.6	3.5	$2,020	$1,706	$285 per case identified
	ALT testing followed by HCV testing in those with elevated ALT values.	Test offered to those at significant risk, according to responses to all the questions, with the exception of socially intrusive questions, of a survey.	12%	$4,292	3.5	$4,292	$1,706	$4,310 per case identified
Leal, 1999	One prevalence round of screening in IDU population	No screening	60%	Not specified		Not specified		£9,300 per QALY gained
Loubiere, 1999	Analysis of 5 screening strategies:1) PCR; 2) ELISA; 3) ELISA followed by ELISA if tested positive; 4)ELISA followed by RIBA if tested positive; 5) Two ELISA test in parallel followed by different treatment strategies		IDUs	Not specified		Not specified		FF 116,168 to 623,354 per case avoided
Loubiere, 2003	‘Wait and treat cirrhosis’–initiation of HCV treatment after cirrhosis (symptomatic)	No HCV screening or treatment, but management of complications (cirrhosis)	IDUs 80%	Not specified		Not specified		Dominated
	Screening via EIA test follows first positive EIA test	No HCV screening or treatment, but management of complications (cirrhosis)	IDUs 80%	Not specified		Not specified		$4,513 per LYG
	Screening via PCR test added to first positive EIA test	No HCV screening or treatment, but management of complications (cirrhosis)	IDUs: 80%	Not specified		Not specified		$4,897 per LYG
McGarry, 2012	Birth cohort screening (general population)	Risk-based screening	Not specified	$792	$529	15.002	14.995	$37,720 per QALY gained
Nakamura, 2008	Screening general population every 5 years:40–49 years	No screening	Genotype 1: 70%Genotype 2&3: 30%	$59,657	$57,409	17.39	14.74	$848 per LYG
	Screening general population every 5 years:50–59 years	No screening	Genotype 1: 70%Genotype 2&3: 30%	$55,530	$51,995	15.92	13.75	$1,627 per LYG
	Screening general population every 5 years:60–69 years	No screening	Genotype 1: 70%Genotype 2&3: 30%	$47,732	$42,948	13.55	12.02	$3,133 per LYG
	Screening general population every 5 years:70 years	No screening	Genotype 1: 70%Genotype 2&3: 30%	$43,640	$37,622	12.13	10.89	$4,825 per LYG
	Screening high risk groups (high level of amino-transferase, major surgery, blood transfusion during child birth)40–49 years	No screening	Genotype 1: 70%Genotype 2&3: 30%	$55,425	$57,409	17.39	14.74	Dominated
	Screening high risk groups 50–59 years	No screening	Genotype 1: 70%Genotype 2&3: 30%	$53,131	$51,995	15.92	13.75	$523 per LYG
	Screening high risk groups60–69 years	No screening	Genotype 1: 70%Genotype 2&3: 30%	$46,456	$42,948	13.55	12.02	$2,297 per LYG
Plunkett, 2008	Routine screening in pregnancy and subsequent treatment (48 week course)	No screening	1%	$4,660	$4,552	54.48947	54.48958	Dominated
	Screening with treatment plus caesarean delivery	No screening	1%	$4,669	$4,552	54.48968	54.48958	$1,170,000 per QALY gained
Rein, 2012	Birth Cohort screening with standard care	Risk based screening, treatment with standard care	Not specified	$327	$246	16.361	16.356	$15,700 per QALY gained
	Birth cohort screening with additional treatment	Risk based screening with standard care	Not specified	$530	$246	16.364	16.356	$35,700 per QALY gained
	Birth cohort screening with additional treatment	Birth Cohort screening with standard care	Not specified	$530	$327	16.364	16.361	$73,700 per QALY gained
Singer, 2001	Initial screening with ELISA, confirmation with PCR–general population	No screening	2.9%	$511	$390	23.594	23.596	Dominated
Stein, 2003	Universal screening of attendees of GUM clinics	No screening	1.5%	Not specified		Not specified		£84,570 per QALY gained
	Screening IDUs (non-current injectors):	No screening	48.6%	Not specified		Not specified		£27,138 per QALY gained
	Selective screening of 10% who present	No screening	9.9%	Not specified		Not specified		£34,288 per QALY gained
	Selective screening of 20% who present	No screening	6.2%	Not specified		Not specified		£39,647 per QALY gained
Stein, 2004	Screening and treating IDUs in one prevalent round of screening	No screening	Not specified	Not specified		Not specified		£28,120 per QALY gained
Sutton, 2006	Verbally screen for ever having received positive HCV test and for ever having injected illicit drugs	No screening	Not specified	£28,192,000	0	13,413	0	£2,102 per case identified
	Verbally screen for injecting illicit drugs only	Verbally screen for ever having received positive HCV test and for ever having injected illicit drugs	Not specified	£30,444,000	£28,192,000	13,548	13,413	£16,625 per case identified
	No verbal screening	Verbally screen for injecting illicit drugs only	Not specified	£53,123,000	£30,444,000	17,098	13,548	£6,388 per case identified
	Verbally screen for past positive HCV test only	No verbal screening	Not specified	£54,670	£53,123,000	16,927	17,098	Dominated
Sutton, 2008	Testing and treatment of people entering prison with possibility of later spontaneous presentation for screening and treatment in a community location	Presentation for screening and treatment only possible in a community location	Not specified	£1,012,509	£737,798	1,650	1,644	£54,852 per QALY gained
Thompson-Coon, 2006	Offer of testing to all those within a target age group (population approach)	Non-case finding approach; individuals present spontaneously for testing	12.5%	£570	£400	2.77	2.26	£15,493 per QALY gained
	Offer of testing to those known to be at highest risk of having contracted HCV (targeted approach)	Non-case finding approach; individuals present spontaneously for testing	49%	£2,357	£1,598	9.05	9.00	£16,493 per QALY gained
Tramarin, 2008	Screening IDUs No stratification by genotype	No screening		€124,860,989	€153,165,347	422,884	413,848	-€3,132/QALY gained Dominance
	Screening IDUs Genotype 1,4	No screening		€90,093,972	€130,231,070	282,763	274,952	-€5,139/QALY gained Dominance
	Screening IDUs Genotype 2,3	No screening		€34,767,017	€22,934,277	140,121	138,896	€9,659/QALY gained
Cipriano, 2012	Anti-HIV upon entry to ORT	No screening	35% among IDUs, 1.7% among non-IDUs.	£1,580,365 (incremental)		169 LYs, 141 QALYs (incremental)		£9,365 per LY, £11,191 per QALY
	Anti-HIV Annual	Upon entry to ORT	35% among IDUs, 1.7% among non-IDUs.	£2,874,166 (incremental)		245 LYs, 206 QALYs (incremental)		£16,938 per LY, £20,075 per QALY
	Anti-HIV 6 months	Annual	35% among IDUs, 1.7% among non-IDUs.	£3,832,733 (incremental)		281 LYs, 237 QALYs (incremental)		£26,436 per LY, 30,713 per QALY
	Anti-HIV+RNA upon entry to ORT	Anti-HIV 6 months	35% among IDUs, 1.7% among non-IDUs.	£5,509,497 (incremental)		337 LYs, 287 QALYs (incremental)		£30,323 per LY, £33,503 per QALY
	Anti-HIV+RNA Annual	Anti-HIV+RNA upon entry to ORT	35% among IDUs, 1.7% among non-IDUs.	£11,200,954 (incremental)		487 LYs, 416 QALYs (incremental)		£37,900 per LY, £44,141 per QALY
	Anti-HIV+RNA 6 months	Anti-HIV+RNA Annual	35% among IDUs, 1.7% among non-IDUs.	£16,207,602 (incremental)		574 LYs, 492 QALYs (incremental)		Dominated, £65,883 per QALY
	Anti-HIV; Anti-HCV Annual	Anti-HIV+RNA 6 months	35% among IDUs, 1.7% among non-IDUs.	£25,652,696 (incremental)		731 LYs, 318 QALYs (incremental)		Dominated, Dominated
	Anti-HIV+RNA 3 months	Anti-HIV; Anti-HCV Annual	35% among IDUs, 1.7% among non-IDUs.	£25,664, 563 (incremental)		668 LYs, 574 QALYs (incremental)		Dominated, £115,429 per QALY
	Anti-HIV+RNA; Anti-HCV–annual, upon entry to ORT	Anti-HIV+RNA 3 months	35% among IDUs, 1.7% among non-IDUs.	£30,938,150 (incremental)		930 LYs, 533 QALYs (incremental)		£44,532 per LY, Dominated
	Anti-HIV+RNA; Anti-HCV– 6 months, upon entry to ORT	Anti-HIV+RNA; Anti-HCV–annual, upon entry to ORT	35% among IDUs, 1.7% among non-IDUs.	£35,936,712 (incremental)		1,017 LYs, 609 QALYs (incremental)		£57,192 per LY, Dominated
	Anti-HIV+RNA; Anti-HCV– 6 months, annual	Anti-HIV+RNA; Anti-HCV– 6 months, upon entry to ORT	35% among IDUs, 1.7% among non-IDUs.	£38,956,858 (incremental)		1,060 LYs, 604 QALYs (incremental)		£71,399 per QALY, Dominated
	Anti-HIV+RNA; Anti-HCV– 3 months, upon entry to ORT	Anti-HIV+RNA; Anti-HCV– 6 months, annual	35% among IDUs, 1.7% among non-IDUs.	£45,390,578 (incremental)		1,111 LYs, 691 QALYs (incremental)		Dominated, £168,600 per QALYs
	Anti-HIV+RNA; Anti-HCV– 3 months, annual	Anti-HIV+RNA; Anti-HCV– 3 months, upon entry to ORT	35% among IDUs, 1.7% among non-IDUs.	£48,410,723 (incremental)		1,154 LYs, 686 QALYs (incremental)		£100,749 per LY, Dominated
	Anti-HIV+RNA; Anti-HCV– 3 months, 6 months	Anti-HIV+RNA; Anti-HCV– 3 months, annual	35% among IDUs, 1.7% among non-IDUs.	£49,421,140 (incremental)		1,156 LYs, 683 QALYs (incremental)		£489,639 per LY, Dominated
	Anti-HIV+RNA; Anti-HCV+RNA– 3 months, annual	Anti-HIV+RNA; Anti-HCV– 3 months, 6 months	35% among IDUs, 1.7% among non-IDUs.	£55,246,297 (incremental)		1,162 LYs, 681 QALYs (incremental)		£905,133 per LY, Dominated
	Anti-HIV+RNA; Anti-HCV+RNA– 3 months	Anti-HIV+RNA; Anti-HCV+RNA– 3 months, annual	35% among IDUs, 1.7% among non-IDUs.	£64,329,321 (incremental)		1,170 LYs, 689 QALYs (incremental)		£1,220,703 per LY, Dominated
Eckman, 2013	Screening of ethnically and gender-mixed population in U.S followed by guideline-based treatment.	No screening	1.4%	$1,111.50	$952.67	20.6873 QALYs	20.6839 QALYs,	$47,267 per QALY
Linas, 2012 *PEG/RBV*	6-mo LFTs/12-mo HCVAb test	Symptom based	9.8%	$ 481,600	$ 479,600	183.36 QALMs	182.84 QALMs	$43,700 per QALY
	3-mo LFTs	6-mo LFTs/12-mo HCV RNA test	9.8%	$ 482,300	$ 481,600	183.43 QALMs	183.36 QALMs	$129,700 per QALY
*PEG/RBV+HCV PI*	6-mo LFTs/12-mo HCVAb test	Symptom based	9.8%	$ 488,300	$483,700	185.74 QALMs	184.81 QALMs	$57,800 per QALY
	3-mo LFTs	6-mo LFTs/12-mo HCV RNA test	9.8%	$ 489,100	$488,300	185.78 QALMs	185.74 QALMs	$229,900 per QALY
Liu, 2013	No Screening plus IL-28B guided triple therapy	No screening plus standard therapy	3%	$5,833,793 (incremental cost)		116 QALYs		$50,417 per QALY
	No screening plus universal triple therapy	No Screening plus IL-28B guided triple therapy	3%	$8,076,805 (incremental cost)		145 QALYs		Dominated
	Risk-based plus standard therapy	No screening plus universal triple therapy	3%	$16,795,805 (incremental cost)		181 QALYs		Dominated
	Risk-based plus IL-28B guided triple therapy	Risk-based plus standard therapy	3%	$ 26,537,268 (incremental cost)		397 QALYs		Dominated
	Risk-based plus universal triple therapy	Risk-based plus IL-28B guided triple therapy	3%	$ 30,282,373 (incremental cost)		450 QALYs		Dominated
	Birth-cohort plus standard therapy	Risk-based plus universal triple therapy	3%	$ 35,369,580 (incremental cost)		483 QALYs		Dominated
	Birth-cohort plus IL-28B guided triple therapy	Birth-cohort plus standard therapy	3%	$ 50,876,459 (incremental cost)		859 QALYs		$60,590 per QALY
	Birth-cohort plus universal triple therapy	Birth-cohort plus IL-28B guided triple therapy	3%	$ 56,843,606 (incremental cost)		950 QALYs		$65,749 per QALY
Miners, 2014	Screening of Pakistani/British Pakistani people registered at GPs in London	No intervention	3.2%	£435	£373	17.762 QALYs	17.759 QALYs	£23,200 per QALY
Ruggeri, 2013	Screening plus treatment	No screening	Age Groups:15–30: 2%,31–45: 6%46–60: 7%>60: 5%	€25,341.07	€15,661.57	27.31 QALYs	25.43 QALYs	€5,171.23 per QALY
Schackman, 2014*Treatment with interferon-containing regimens*	Off-site referral	No intervention	0.4%	$109,020	$108,900	16.546 QALYs	16.542 QALYs	Dominated
	On-site rapid HCV	Off-site referral	0.4%	$109,290	$109,020	16.563 QALYs	16.546 QALYs	$18,300 per QALY
	On-site rapid HCV and HIV	On-site rapid HCV	0.4%	$109,430	$109,290	16.565 QALYs	16.563 QALYs	$64,500 per QALY
*Treatment with SOF-based regimens*	Off-site referral	No intervention	0.4%	$110,850	$110,660	16.605 QALYs	16.599 QALYs	Dominated
	On-site rapid HCV	Off-site referral	0.4%	$111,390	$110,850	16.632 QALYs	16.605 QALYs	$22,000 per QALY
	On-site rapid HCV and HIV	On-site rapid HCV	0.4%	$111,540	$111,390	16.634 QALYs	16.632 QALYs	$64,000 per QALY
*Treatment with hypothetical interferon-free regimens*	Off-site referral	No intervention	0.4%	$113,710	$113,420	16.654 QALYs	16.645 QALYs	Dominated
	On-site rapid HCV	Off-site referral	0.4%	$114,680	$113,710	16.691 QALYs	16.654 QALYs	$27,100 per QALY
	On-site rapid HCV and HIV	On-site rapid HCV	0.4%	$114,820	$114,680	16.694 QALYs	16.691 QALYs	$64,300 per QALY
Urbanus, 2013	Screening–all pregnant women	No screening–all pregnant women	0.2%	€55,474	€13,605	0.8 LYs	0	€52,472 per LYG
	Screening–non-Western women	No screening–non-Western women	0.43%	€106,307	€28,725	1.65 LYs	0	€47,113 per LYG
	Screening–all pregnant women with addition of protease inhibitors to standard treatment for all genotypes	No screening–all pregnant women	0.2%	Not specified		Not specified		€88,162 per LYG
	Screening–non-Western women with addition of protease inhibitors to standard treatment for all genotypes	No screening–non-Western women	0.43%	Not specified		Not specified		€86,005 per LYG
Wong, 2015*Age 26–64*	Screen and treat with pegylated interferon plus ribavarin	No screening	Age 25–34 and 35–44: 0.4%, 45–54 and 55–64: 0.8%	$71,450	$71,327	13.7685 QALYs	13.7653 QALYs	$38,117 per QALY
	screen and treat with pegylated interferon and ribavarin–based direct-acting antiviral agents	No screening	Age 25–34 and 35–44: 0.4%, 45–54 and 55–64: 0.8%	$71,593	$71,327	13.7729 QALYs	13.7653 QALYs	$34,783 per QALY
	Screen and treat with interferon-free direct-acting antivirals.	No screening	Age 25–34 and 35–44: 0.4%, 45–54 and 55–64: 0.8%	$71,593	$71,327	13.7716 QALYs	13.7653 QALYs	Dominated
*Age 45–64*	Screen and treat withpegylated interferon plus ribavarin	No screening	Age 25–34 and 35–44: 0.4%, 45–54 and 55–64: 0.8%	$83,476	$83,335	12.1068 QALYs	12.1027 QALYs	$34,359 per QALY
	screen and treat with pegylated interferon and ribavarin–based direct-acting antiviral agents	No screening	Age 25–34 and 35–44: 0.4%, 45–54 and 55–64: 0.8%	$83,672	$83,335	12.1104 QALYs	12.1027 QALYs	$55,151 per QALY
	Screen and treat with interferon-free direct-acting anti-virals.	No screening	Age 25–34 and 35–44: 0.4%, 45–54 and 55–64: 0.8%	$83,673	$83,335	12.1122 QALYs	12.1027 QALYs	$36,471 per QALY

Studies evaluating cost-effectiveness of screening in the general population were carried out in a range of countries and healthcare settings including hospital or secondary care [25, 28], primary care [29, 38, 50] or community settings [34, 47]. Screening interventions often combined different strategies, including combinations of ELISA and PCR testing [26, 29] and testing individuals with increased ALT levels [25]. Most studies evaluated a one-off screening intervention, with the exception of one study that analysed screening every five years [28]. Linas et al evaluated ten screening strategies, including symptom-based screening and liver function tests (LFTs) at various intervals combined with HCV Ab tests [52]. Comparators were either no screening or the status quo [26, 28, 29, 37, 50, 51] or strategies were compared with each other [25, 38]. More recent studies have concentrated on birth cohort screening in the US [32, 33, 35], evaluating the cost-effectiveness of one-off screening for a cohort born between 1946 and 1970 [32], a cohort born between 1945 and 1965 [33] and all adults aged 40 to 74 year, who are unaware of their HCV infection status [35]. HCV prevalence in this population was comparatively high. The initiation of a one-off screening intervention was assessed and compared with current risk-based screening interventions.

Most studies used a decision tree to reflect the screening arm of the intervention, followed by a Markov model to present the disease module [26–29, 31, 32, 34–36, 47, 51]. Some studies employed a Markov model only [33, 36, 37, 50] and two studies did not use a model to evaluate the cost-effectiveness of screening for HCV [25, 38].

Conclusions about cost-effectiveness of individual screening strategies were mixed for the general population. This reflects the evolving approaches to treatment and management over time. Treatment regimes have been changing rapidly over the last years and recent models have used interferon-free DAA treatments [37], so when interpreting results, a distinction needs to be drawn between main treatment strategies, i.e. IFN/RBV models, triple therapy (IFN/RBV+telaprevir or boceprevir) and IFN-free DAAs. Apart from new treatment regimes, recent studies in the US showed birth cohort screening to be cost-effective when compared with risk-based screening, but only one of these was able to include these new treatment options [35] and the remaining studies included standard treatment. However all studies showed favourable cost-effectiveness results which are mainly due to the very high prevalence in this particular birth cohort in the US. People will have lived with undetected HCV infection for a long time, so will be at a more advanced stage of infection, when detected, rendering treatment to be more effective. In general, the results of the studies were found to be sensitive to assumptions made on prevalence in the general population, and discount rates applied to future costs and benefits. In addition to the underlying prevalence of HCV infection, studies identified the annual rate of progression from chronic HCV to cirrhosis as an important factor that substantially influenced cost-effectiveness results as well as the loss of quality of life associated with the knowledge of HCV infection.

A total of ten papers evaluated cost-effectiveness of screening IDUs; four in the UK [24, 40, 41, 44], two in France [26, 27]; one in Italy [45] and three in the US [38, 46, 47]. Settings included primary care, prison services and substance abuse treatment centres. Screening was performed as one-off screening in all studies, with a strategy of ‘no screening’ frequently serving as the comparator. Thompson-Coon et al analysed two different approaches within a former IDU population; a population approach and a targeted approach [44]. Tramarin et al evaluated the cost-effectiveness of screening, and compared two strategies which followed the incident IDU population over time using two scenarios, chronic and acute infection [45]. More recently, Cipriano et al evaluated screening IDUs for both, HCV and HIV infection simultaneously [46]. Schackman et al also investigated various screening strategies, one of them also combining HCV and HIV screening of IDUs [47]. The majority of studies employed a decision tree followed by a Markov model [24, 26, 27, 41, 44, 47]; one study used a Markov model only [45] and Cipriano et al employed a dynamic model [46]. Leal et al used a decision tree analysis [40]. Honeycutt et al did not use an economic model and presented an average cost-effectiveness ratio per person who returned to receive their results using a multiplicative formula [38].

Two UK studies reported screening and treating IDUs in one prevalent round of screening to be cost-effective compared to no screening, in a UK context. However, there were substantial differences in estimated ICERs– £28,120 per QALY gained using a decision tree followed by a Markov model and discounting of costs and benefits [41] and £9,300 per QALY gained based on a decision tree analysis of a hypothetical cohort of IDUs only discounting future costs [40]. Studies concluded that screening is likely to be most cost-effective in individuals whose infection is more long-standing and who would be at higher risk of progression [24] [44]. Tramarin et al evaluated screening strategies for different genotypes and concluded that screening dominated the no screening strategy for genotypes 1 and 4, but for genotypes 2 and 3, screening would cost €9,659 per QALY gained compared to no screening [45]. Similar to the general population, faster progression rates were found to result in more favourable cost-effectiveness ratios, mainly due to benefits being incurred much earlier [43]. In general, studies were in agreement that screening IDUs in various settings (mainly prison) is cost-effective compared to a strategy of no screening. Cost-effectiveness results were sensitive to the assumptions on prevalence of HCV, in particular for the difficult to treat genotypes, and on progression rates.

Using interferon-free regimens as one of their treatment options, Schackman et al estimated an ICER of $27,100 per QALY gained for rapid on-site testing for HCV compared to no testing and an ICER of $64,300 per QALY gained for rapid on-site testing for both, HCV and HIV compared to on-site rapid HCV testing alone [47].

Loubiere et al assessed cost-effectiveness of five strategies of varying tests of screening blood recipients and combined these with two different treatment strategies that would follow after a positive diagnosis [27]. Jusot and Colin compared the cost-effectiveness of screening for HCV with no screening in recipients of blood donations [48]. A decision tree followed by a Markov model was employed in two studies [26, 27]. A similar approach to the decision analytical model was used by Jusot and Collin [48]. The studies were in general agreement that screening blood recipients for HCV infection was unlikely to be cost-effective as the high prevalence in this population is off-set by higher mortality rates.

Two studies compared strategies for screening women during pregnancy [49, 50]. Plunkett et all compared three strategies: (i) routine screening followed by a 48-week course of treatment; (ii) routine screening followed by a 48-week course of treatment and elective caesarean delivery; (iii) usual care (no screening) [49]. The evaluation was based on a decision tree supplemented by a Markov model. Screening followed by treatment was found to be dominated (more costly and less effective) by usual care. Similarly, screening followed by treatment plus elective caesarean delivery was shown not to be cost-effective when compared with usual care (ICER of $1,170,000 (USD) per QALY gained). The ‘no screening’ strategy continued to dominate even when a higher prevalence (up to 10%) was assumed. Urbanus et al evaluated screening strategies for two groups in the Dutch setting: i) all pregnant women and ii) first-generation pregnant migrants from non-Western countries [50]. The authors based their evaluation on a Markov model and found that screening followed by standard treatment for all pregnant women would cost €52,473 per LYG compared to no screening. Screening non-Western pregnant women, followed by the same treatment regime, would cost €47,113 per LYG. When protease inhibitors were added to standard treatment, screening was found not to be cost-effective in a Dutch setting with ICERs of €88,162 and €86,005 per LYG [50].

Screening of HCWs was evaluated comparing four strategies (‘French recommendation’, ‘European recommendation’, ‘baseline US recommendation’ and ‘alternative US recommendation’) following occupational exposure in France [53]. Both, the European and French strategy, were dominated by other strategies in the analysis. Compared with the baseline US strategy, the alternative US strategy showed an ICER of €2,020 per QALY gained.

Linas et al evaluated screening HIV infected MSM for HCV using a combination of different screening strategies: symptom-based screening and liver function tests (LFTs) at various intervals with/without HCV Ab tests [52]. Employing a Monte Carlo simulation model (‘HEP-CE’) to estimate cost-effectiveness of screening, the authors compared each strategy to symptom-based screening alone. They concluded that screening for acute HCV in HIV infected MSM using six-monthly LFTs and a 12-months HCV Ab test would cost $43,700 per QALY gained compared to symptom-based screening combined with IFN/RBV. Adding protease inhibitors to this strategy resulted in an ICER of $57,800 per QALY gained [52].

A recent study from the UK evaluated cost-effectiveness of screening migrants from the Indian sub-continent [51]. Participants were invited via an opt-out strategy and subsequently invited for screening. The study employed a Markov model to evaluate cost-effectiveness and concluded that opt-out GP case finding was potentially cost-effective with an ICER of £23,200 per QALY gained compared to no intervention, but there was a substantial degree of uncertainty surrounding these estimates. Increasing the treatment costs and adding boceprevir or telaprevir to treatment did not change these results [51].

### Critical appraisal of economic models–Hepatitis C Virus

**Fig 6:**
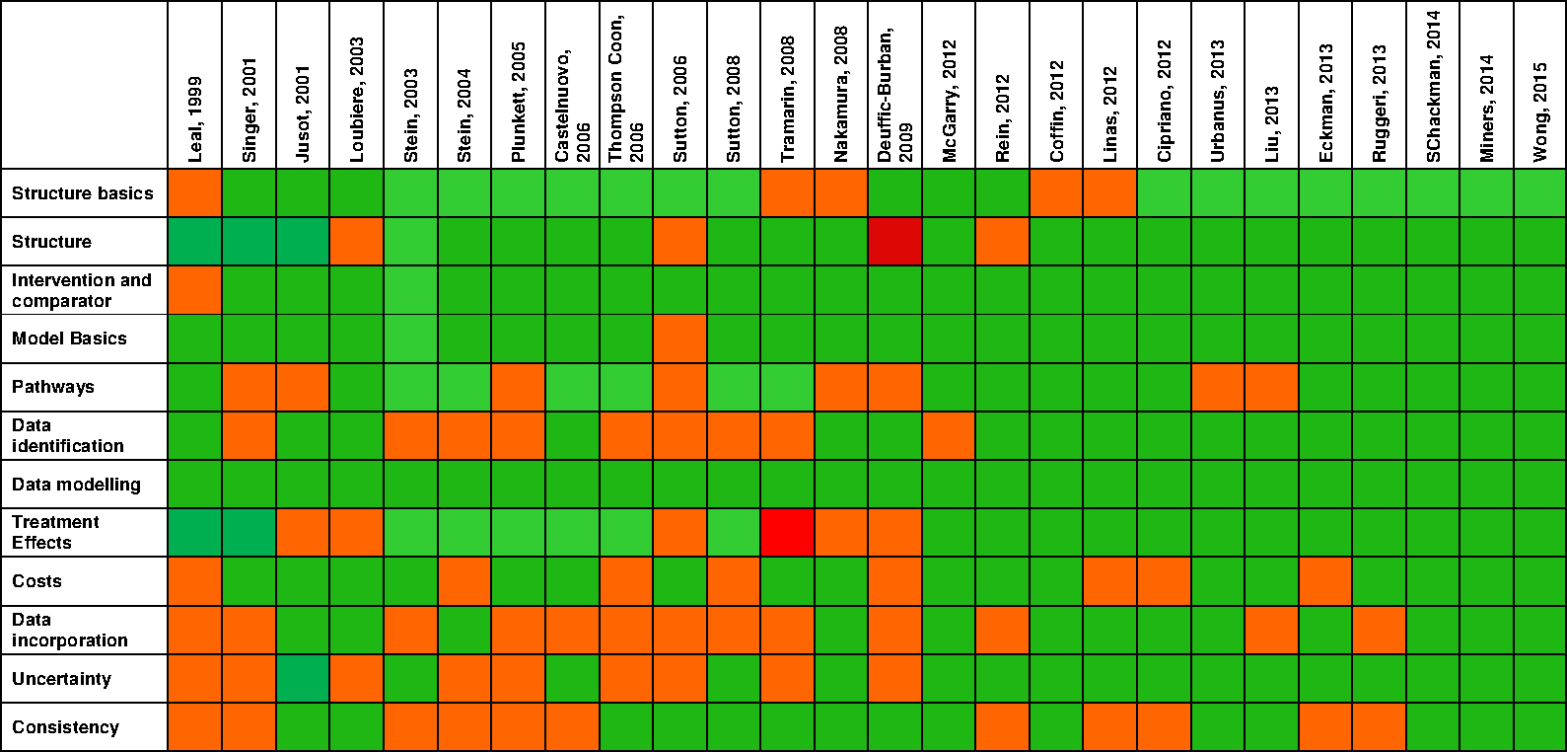
Performance Matrix—HCV screening models.

The checklist developed by Philips et al was completed for all studies that used an economic model to estimate cost-effectiveness of screening (n = 26). The associated performance matrix (Fig 6) shows results for each individual study and their performance in each of the twelve distinct categories of the checklist. The pattern in the matrix shows that those studies published more recently performed better. Model inputs were found to be consistent with the stated perspective of the model; however, some studies broadly claimed to have adopted a societal perspective, while only considering direct medical costs. In general, studies provided a clear presentation of the model structure with only a few studies presenting just the Markov part of the model. Similar to some HBV studies, one major limitation relates to the way ICERs were estimated. When multiple strategies were evaluated, although all studies presented incremental analyses for synthesising costs and benefits, some studies presented a series of pairwise comparisons against a common comparator, for instance the status quo or no screening, rather than a fully incremental analysis.

Although the majority of studies employed discounting for costs and effects, the choice of rates was frequently not justified. In most cases rates would be chosen according to current policy guidelines and, as a result, authors may not have felt the need to provide additional justification. Studies with a short time horizon (< 1 year) did not apply any discounting. In general, studies performed well against the requisite criteria. With regard to sensitivity analysis, the majority of studies employed one-way sensitivity analyses to assess parameter uncertainty.

Broadly, cost-effectiveness of screening for HCV was relatively insensitive to variations in most of the input parameters, such as the cost of screening and treatment. However, Urbanus et al showed that adding protease inhibitors to standard treatment regimes altered conclusions about the cost-effectiveness of screening pregnant women [50]. Overall, the parameter that cost-effectiveness results were most sensitive to was the prevalence in the target population, including varying prevalence rates in different genotypes. For general population studies, assumptions on prevalence rates varied from 0.9% (women) [38] to 6.5% (men) [25]. Prevalence estimates for IDUs ranged from 12.5%[44] to 80% [26] and for blood transfusion recipients rates varied between 3% [48] to 7% [25]. The prevalence rate that was used for evaluating screening in migrants was 3.2%, which was varied between 1% and 5% in sensitivity analyses [51], and 9.8% was used by Linas et al, to evaluate cost-effectiveness of screening HIV positive MSM for HCV [52]. Prevalence of HCV infection in HCWs has not been used as a parameter in the analysis; however this is most likely to be reflected in transmission risk from patients to HCW during occupational exposure. Several studies showed in sensitivity analyses that their cost-effectiveness results changed considerably when the prevalence rate was varied. Stein et al showed that estimated costs per QALY increased rapidly once the underlying prevalence in the population likely to attend GUM clinics decreased below 3% [37].

Cost-effectiveness results are also sensitive to the assumptions made on prevalence of HCV for the difficult to treat genotypes and to progression rates from chronic HCV to cirrhosis and Singer et al found that for screening to be cost-effective this rate should be greater than 2.5% [36]. Another important factor that would impact on cost-effectiveness results was the loss of quality of life (disutility of knowledge of HCV infection) which was assumed to be 0.02 in the baseline scenario. Even for a disutility of 0.01, the ‘no screening’ option remained the preferred strategy [36]. Furthermore, the staging of liver disease was found to impact on cost-effectiveness results. Identifying individuals with more advanced infection yielded more favourable cost-effectiveness results than screening and detecting those in the early stages of the disease. Variation in the discount rate had a considerable impact on cost-effectiveness results. Further assumptions, although less important in terms of cost-effectiveness results, were made on transmission risks, in particular in those studies evaluating screening strategies for pregnant women.

The associated PRISMA checklist can be found in the S3 Table.

## Discussion

For both, HBV and HCV screening, studies used various economic models in order to derive their cost-effectiveness conclusions, but the overall approach was relatively consistent across studies. For example, disease states represented in the various models did not differ fundamentally and the overall model structure was found to be consistent. Where models were found to differ, however, was the screening component which varied between studies depending on interventions examined, such as universal screening and targeted or risk-based screening. Models were also found to vary in assumptions made on the input parameters, population, and outcome measures to express cost-effectiveness results. The critical clinical input parameters that were identified include: prevalence, test sensitivity and specificity and treatment effectiveness. Most studies focussed on direct medical costs adopting a healthcare payer perspective, while some studies also took account of costs associated with loss of productivity, adopting a societal perspective. The population groups evaluated did not show any major gaps in terms of certain risk-groups that were not included. On the contrary, the review showed that some of the populations studied in the past should not be the focus of future research as there is evidence that screening does not offer a cost-effective result- this is the case in particular for HBV screening, where there was very little recent literature and the existing evidence suggests that it might only be a cost-effective strategy in migrant populations and not in other population groups, such as the general population. This overall result was not altered by the use of different economic models. Various outcome measures were used, including QALYs, life-years gained, the number of cases detected and the number of infections prevented. Discount rates varied, both between countries in which the studies were carried out, but also within countries and over time. Any results obtained from older studies, applying discount rates that have been updated since, should therefore be interpreted with care.

The treatment landscape for HCV has been changing dramatically. With the advent of direct acting antiviral therapy in 2008/09, first generation interferon-containing regimens substantially increased the efficacy in genotype 1 patients [54]. Even more effective, shorter duration, more tolerable, and interferon-free regimes have recently become available. These new treatments come at a higher cost but are generally believed likely to increase treatment uptake. Earlier studies have modelled their costs and outcomes on the basis of Interferon + Ribavirin or Peg INF/Ribarivin, while more recent studies will have modelled on the basis of Peg INF + Ribavirin and DAAs. The most recent studies are now looking at IFN-free DAAs with Wong et al showing that screening and treatment using IFN-free DAAs would likely be cost-effective [37]. Thus, the focus should be on those more recent studies, which applied the latest treatment regimes, test methods and had more data on which to base their models. Findings from older publications need to be interpreted with caution as there could be a potential relationship between the age of the study and the assumed treatment effectiveness and cost.

Studies have pre-dominantly used static models, not accounting for the dynamic transmission element of the infection and any potential treatment as prevention benefit, in particular for HCV [24–29, 38–41, 43, 48, 53]. Studies employing a static Markov model structure include progression of the disease in an individual, and either no re-infection or re-infection at a fixed rate through time irrespective of population prevalence. The use of a dynamic transmission model assumes the risk of an individual’s infection is related to the population prevalence, which may change through time with screening and treatment [46]. The use of a dynamic model increases the complexity, but allows for the inclusion of the possible population benefits of screening and treatment on transmission, in addition to the individual benefits. However, dynamic models require an understanding of the disease epidemic and modes of transmission.

## Conclusions

Overall, published modelling strategies for HBV and HCV screening do not differ fundamentally. Future models evaluating the cost-effectiveness of screening for HBV and HCV infections should concentrate on selection of the most relevant input parameters, (i.e. those to which cost-effectiveness results are most sensitive) and the selection of those population groups for which screening has been shown to be cost-effective. In addition to prevalence, understanding disease incidence is important as it impacts on the frequency of testing high-risk populations. Careful consideration of dynamic versus static modelling is also recommended. A dynamic model structure includes benefits of the prevention of the transmission from either cured individuals or benefits of averted transmission due to knowledge about their infection. It therefore accounts for direct effects on the infected individuals and also indirect effects of protecting other persons, such as sexual or injecting partners, in the future. Hence, future research should concentrate on these methodological issues. In particular, more consideration should be given to the evaluation of screening strategies for multiple infectious diseases, such as HCV and HIV simultaneously as well as birth-cohort screening. Birth-cohort screening has shown a favourable ICER compared to risk-based screening, mainly due to the fact that individuals will have lived with the infection being undetected for a number of years, so that treatment will be more effective in these advanced stages of infection. So far, these have only been assessed in a US context, and the evaluation of birth-cohort screening in a European context would be valuable as the age structure might differ.

## Supporting Information

S1 Table Evidence Summary HBV. (DOCX)

S2 Table Evidence Summary HCV. (DOCX)

S3 Table PRISMA Checklist. (DOCX)

S1 Text Search Strategy. (DOCX)

S2 Text Methods for Model Critique (Categories of Assessment based on the Philips Checklist). (DOCX)
