# Supplementary material for: Cost-Effectiveness of HBV and HCV Screening Strategies – A Systematic Review of Existing Modelling Techniques
Source: PLoS One. 2015 Dec 21;10(12):e0145022. doi: 10.1371/journal.pone.0145022 (PMC4686364; doi:10.1371/journal.pone.0145022)
Supplement: S1 Table — (DOCX) [file pone.0145022.s001.docx]

**S3 Table - Evidence Summary HBV**

| **Study Details** | **Population** | **Intervention/Comparator** | **Methods** |
| --- | --- | --- | --- |
| Antonanzas et al; 1995 [[21](#_ENREF_21)]  Spain  Spanish Pesetas (price year 1992)  **Study conclusion:**  Mass adolescent vaccination was associated with the lowest cost per infection avoided | Newborn infants and adolescents  **Prevalence:**  Varying prevalence according to age groups.    Two different assumptions of infection rates used:  A – constant annual infection rate of 1.5% until age 15, increased during adolescence and returned to lower levels when aged 20-32 years.  B – risk distributed among different age groups (0.14% in ≤14 years, 0.8% in 15-24 years; 0.53% 25-31 years) | **Intervention:**  Primary prevention and screening:  (1) mass immunisation of adolescents  (2) mass immunisation of infants  (3) combined mass immunisation  (4) combined strategies – screening all women during pregnancy for HBV surface antigen combined with the above strategies  **Comparator:** ‘do nothing’  **Setting:** Community and hospital | **Time Horizon:** 20 years after vaccination  **Perspective**: not stated  **Compartmental and Markov models**  **Clinical data:** derived from published literature (single study) and expert opinion  **Costs:**  Fixed costs (information campaign for health professionals and general population, health education, sero-epidemiological surveys and research) and variable costs (from Catalonia Health Department: 3 vaccine doses, injection equipment and labour time plus cost of booster dose)  Discounted at 5%.  **Outcome measures:**  Number of infections avoided; not discounted. |
| Arevalo and Washington; 1988 [[13](#_ENREF_13)]  US  US dollars (price year 1985)  **Study conclusion:**  Screening of women during pregnancy and vaccination of infants of test-positive mothers would be cost-effective; the remains the case at a prevalence rate of 0.06% | Women during pregnancy  **Prevalence**:  0.2% (resents prevalence of the US population)  Other prevalence rates were also tested (representing medical personnel; CDC recommended screening groups; very high-risk groups; highest-risk groups) | **Intervention:** screening (HBsAg) and vaccination (HBIG at birth and repeated hepatitis vaccine at months 1 and 6)  **Comparator:** no screening  **Setting**: not specified | **Time horizon:** lifetime  **Perspective:** health system  **Decision Tree**  **Clinical Data:** prevalence, test specificity and sensitivity, neonatal transmission probability, vaccine efficacy, probabilities of acute hepatitis in neonates and chronic liver disease (published literature and expert opinion using the DELPHI method)  **Costs:**  Direct costs (screening, vaccination, physician visit, test, care for neonatal HBV and chronic liver disease, hospitalisation costs)  Indirect costs**:** loss of productivity.  Discounted at 4% (6%)  **Outcome measures:**  Case prevented |
| Bloom et al; 1993 [[8](#_ENREF_8)]  US  US dollars (price year 1989)  **Study conclusions:**  HBV vaccine is most cost-effective when a strategy of screening newborns is combined with routine administration to 10-year old children | General population:  (1) Newborns  (2) 10-year old adolescents  (3) high-risk adult population  (4) the general adult US population  (5) high-risk newborns and adolescents | **Interventions**:  (i) Screen  (ii) Vaccinate  **Comparator:**  No intervention  **Setting:** not specified | **Time Horizon:** 30 years (three consecutive 10 year periods)  **Perspective:** payer  **Decision tree**  **Clinical data:** compliance and vaccine effectiveness, epidemiologic parameters and disease management profile (critical review of the literature and expert opinion)  **Costs:** obtained from actual private insurer payments adjusted for out-of pocket expenditures. Discounted at 5%.  **Outcome measure:**  Life years saved; not discounted and at 5%. |
| Eckman et al; 2011 [[9](#_ENREF_9)]  US  US dollars (price year 2008)  **Study conclusions:**  Screen and treat with low cost nucleoside or nucleotide was found to be the most cost-effective strategy.    Screening based on the 2% prevalence threshold for chronic HBV infection according to current US CDC recommendation is shown to be cost-effective. | General population: hypothetical 35 year old man  **Prevalence**:  0.4%  A range of population prevalence were also tested | I**nterventions:**  Screening for HBV, plus 4 treatment options:  (1) pegylated interferon-alpha 2a for 48 weeks  (2) low cost nucleoside or nucleotide that had high rate of development of viral resistance for 48 weeks  (3) prolonged treatment with low-cost, high resistance nucleoside or nucleotide, followed by salva salvage therapy with high-cost, low-resistance nucleoside or nucleotide, for those, who developed resistance  (4) prolonged treatment with high-cost, low resistance nucleoside or nucleotide  **Comparator:** no screening  **Setting:** Primary Care | **Time Horizon:** lifetime  **Perspective:** societal  **Decision Tree plus Markov model**  **Clinical data:** Published literature.  Discounted at 3%  **Outcome measures:**  QALYs  Discounted at 3% |
| Hutton et al; 2007 [[16](#_ENREF_16)]  US  US dollars (price year 2006)  **Study conclusions:**  Screening programs for HBV among Asian and Pacific Islander adults are likely to be cost effective. | Immigrants: hypothetical cohort of 10,000 Asian and Pacific Islander adults in the US aged 20 to 60 years  **Prevalence**:  10% (range of 4.3% to 14.5% tested) | (i) Status quo (i.e. voluntary screening only and no incremental screening or vaccination) (ii) Universal vaccination  (iii) Screen and treat  (iv) Screen, treat and vaccinate  (v) Screen treat and ring vaccinate (include screening of close contacts) | **Time Horizon:** lifetime  **Perspective:** societal  **Decision Tree and Markov Model**  **Clinical data:** compliance and vaccine effectiveness and epidemiological parameters (published literature and expert opinion)  **Costs:** test costs, vaccine costs and annual HBV medical management costs. Discounted at 3%  **Outcome measures:**  Deaths averted and QALYs. Discounted at 3% |
| Kim et al 2006 [[10](#_ENREF_10)]  US  US dollars (price year 2000)  **Study conclusions:**  Routine provision of Hepatitis B vaccine at major HIV CTSs would be a highly effective and cost-effective approach to preventing Hepatitis B among high-risk adults | General population:  two hypothetical cohorts of high-risk adults, aged 20 to 49 years attending (1) counselling and testing sites and (2) STD clinics for HIV testing. | (i) No intervention  (ii) Routine vaccination  (iii) Screening and vaccination  (iv) Screening with initial dose  **Settings:** CTSs and STD clinics | **Time Horizon:** 45 to 75 years  **Perspective:** societal  **Decision tree and Markov model**  **Clinical data:** vaccine efficacy, epidemiological parameters, vaccine acceptance (synthesised from literature)  **Costs:** Direct costs (program costs, treatment costs). Discounted at 3%  **Outcome measures:** Cases of HBV prevented, QALYs, life-years saved. Discounted at 3% |
| Kwan-Gett et al; 1994 [[22](#_ENREF_22)]  US  US dollars (price year 1993)  **Study conclusions:**  Based on cost per rate of complete vaccination, prevaccination testing is not cost-effective in this population. | Preadolescents and adolescents: hypothetical cohort of 100,000 11-year old children presenting for well-child care | 1. Prevaccination testing for hepatitis B surface antibodies 2. No testing 3. Testing at the same time as the first vaccine dose   **Setting:** children attending the well-child care clinic for measles, mumps and rubella vaccine booster | **Time Horizon:** not stated  **Perspective:** not stated; possibly societal given the inclusion of indirect costs  **Decision tree**  **Clinical data:**  Compliance rate and seroprevalence rate (published literature)  **Costs:**  Direct costs (testing, vaccination) and indirect costs (loss of earnings of mothers due to clinic attendance)  **Outcome measures:**  Cost per patient protected from HBV. No discount rates applied |
| Mulley et al; 1982 [[11](#_ENREF_11)]  US  US dollars (price year 1980)  **Study conclusions:**  Screening followed by vaccination of homosexual men and vaccination without prior screening of surgical residents would result in savings of medical costs. | General population: (1) homosexual men,  (2) surgical residents  (3) general population | (i) Vaccinate all  (ii) Screen and then vaccinate only those without evidence of HBV immunity  (iii) Neither screen nor vaccinate, but respond to HBV exposure with passive immunisation | **Time Horizon:** 20 years  **Perspective**: not stated  **Decision tree**  **Clinical data:** vaccine efficacy and safety, sensitivity and specificity of test, rate of accidental exposure, probabilities of sequelae (literature and expert opinion)  **Costs:**  Physician time, diagnostic tests costs, hospitalisation costs. Discounted at 6%.  **Outcome measures:**  Case of hepatitis B prevented. No discount rate specified. |
| Rein et al; 2011 [[17](#_ENREF_17)]  US  US dollars (price year not stated, but cost data were collected between July 2008 and January 2009)  **Study conclusion:**  The cost effectiveness of integrating screening into community programmes should be explored. | Immigrants: mainly foreign-born people in the US from a single observational study (exploratory pilot study) that was funded to collect information on five different screening programmes.  **Prevalence**:  1.7% for US born participants; 6.3%for foreign born participants | **Interventions:**  (i) Community Clinic model (ii) Community outreach model  (iii) Outreach partnership  (iv) Partnership contract model  Note: Model was used as a term to describe type of screening program  **Comparator:** data provided can be used to compare different models to each other.  **Setting:** Community clinic, community no clinic settings, community events | **Time Horizon:** not stated, 6 months data collection period  **Perspective:** not stated  No model  **Clinical data:**  Data came from observational study of five community-based organisations  **Costs:**  Labour costs, costs of test  **Outcome measures:**  Cost per positive case identified, cost per newly identified positive case, cost per complete screen. No discount rates. |
| Rossi et al; 2013 [[20](#_ENREF_20)]  Canada  Canadian dollars (price year 2011)  **Study conclusion:**  Screening for chronic HBV in adult immigrants soon after arrival was found to be reasonably cost-effective. | Hypothetical cohort of 250,000 new Canadian immigrants  **Prevalence:** 6.5%  A range of population prevalence rates were also tested | Four screening strategies:  i) universal vaccination,  ii) screening for prior immunity and vaccination, iii) chronic HBV screening and treatment  iv) combined screening for chronic HBV and prior immunity, treatment and vaccination  **Comparator:** Status quo (no targeted screening or vaccination)  **Setting:** not specified | **Time Horizon:** Lifetime  **Perspective**: Societal  **Decision Tree plus Markov model**  **Input parameters:** Published literature  **Costs:** Direct costs (program costs, vaccine costs), indirect costs (patient out-of-pocket costs, family care time), productivity costs (lost time at work)  **Outcome measures:** QALYs  **Discount rate:** 3% for costs and outcomes |
| Ruggeri et al; 2011 [[12](#_ENREF_12)]  Italy  Euros (price year 2006)  **Study conclusion:**  Anti-HBV test to all high-risk categories should be highly recommended. | General population**:**  patients at risk ( immigrants from Africa, South-Eastern Asia, South America, Eastern Europe (not Hungary); IDUs, prisoners, individuals with other infections, patients undergoing dialysis, women during pregnancy, subjects with high transaminase).  **Prevalence:**  7% | 1. Screen and treat 2. No screen   **Setting:** local health unit | **Time Horizon:** 40 year  **Perspective**: third party payer  **Decision tree plus Markov model**  **Clinical data:** resistance to treatment, health utilities of health states, transition probabilities in the disease progression pathway (literature). Discounted at 3%.  **Outcome measures:**  QALYs discounted at 3%. |
| Thomas et al; 1990  Australia  Australian Dollars (price year 1988)  **Study conclusions:**  For the patients of the clinic in the study, universal screening and vaccination of all babies of high-risk group mothers are considered appropriate. | Women during pregnancy  Screening strategies targeted different populations: High risk, low risk and the entire group. | 1. Universal screening 2. Screening of high-risk women only 3. Screening of low-risk women only   **Setting:** antenatal clinic | **Time Horizon:** not stated  **Perspective:** not stated  **No model**  **Clinical data:** data obtained from a single study  **Costs:** costs of test. No discount rate presented.  **Outcome measures:**  Number of carriers identified. No discount rate presented |
| Tormans et al; 1993 [[15](#_ENREF_15)]  Belgium  Belgium Francs (price year 1991)  **Study conclusions:**  A prevention campaign against Hepatitis B which screens women during pregnancy for the presence of HBsAg and vaccinates neonates at risk is not cost saving within the Belgian context. | Women during pregnancy  **Prevalence:**  0.67% (weighted average of prevalence observed among varying ethnic groups) | **Intervention**:  Screening and vaccination  **Comparator:**  Doing nothing  **Setting:** Set in outpatient and inpatient care in Belgium. | **Time Horizon:** not specified  **Perspective:** not specified  **Decision tree**  **Clinical data:** vaccine coverage and compliance rates, epidemiological parameters (literature)  **Costs**: direct healthcare costs for screening and vaccinating neonates at risk; treatment costs, hospital and ambulatory costs. Discounted at 5%  **Outcome measures:**  Life-years gained discounted at 5%. |
| Veldhuijzen et al; 2010 [[18](#_ENREF_18)]  The Netherlands  Euros (price year)  **Study conclusions:**  Early detection and treatment of people with HBV infection can have a large impact on liver-related health outcomes. Systematic screening for chronic HBV infection among immigrants is likely to be cost effective. | Immigrants: from intermediate and high endemic countries to the Netherlands  **Prevalence**:  3.35% | **Intervention:**  One-off systematic screening and treatment of eligible patients  **Comparator:**  Status quo: no screening  **Setting:** primary and secondary care | **Time Horizon:** lifetime  **Perspective:** health system  **Markov model**  **Clinical data:**  progression probabilities from literature  **Costs:** direct costs of screening programme, consultation, diagnostic tests, medical management and therapy. Costs of lab tests and medical care based on data from Dutch HC Authority. Entecavir costs from Dutch HC Insurance Board. Discounted at 3%.  **Outcomes:**  QALYs. Discounted at 3%. |
| Wong et al; 2006 [[19](#_ENREF_19)]  Canada  Canadian dollars (price year 2008)  **Study conclusions:**  A selective Hepatitis B screening programme targeted at all immigrants in Canada is likely to be moderately cost-effective. | Immigrants: hypothetical cohort of individuals born outside Canada  **Prevalence**:  4.81% | (i) No screening  (ii) Screen and treat  (iii) Screen, treat and vaccinate | **Time Horizon:** lifetime  **Perspective:** payer  **Decision tree (presumably) and Markov model**  **Clinical data:** probabilities related to acute infection, annual transition to chronic infection and treatment transition probabilities (published literature, literature reviews and official Canadian sources)  **Cost data:** direct medical costs (screening, vaccination and treatment) from literature. Discounted at 5%.  **Outcome measures:**  QALYs. Discounted at 5%. |
| Zurn et al; 2000 [[23](#_ENREF_23)]  Switzerland  Swiss Franc (price year 1996)  **Study conclusions:**  Incremental cost-effectiveness ratios are lower with universal vaccination strategies than with selective vaccination strategies**.** | Women during pregnancy**:** a birth cohort of 85,000 individuals  **Prevalence**:  HBsAg+ prev: 0.5%  HBe+ prev: 10%  Global prevalence: 5% | **Interventions:**  (i) Systematic prenatal screening and vaccination of newborns at risk (ii) Universal vaccination of infants  (iii) Universal vaccination of school children  (iv) Universal vaccination of infants and school children (v) Universal vaccination of infants, school children and adolescents  **Comparator:** Baseline strategy of vaccinating high-risk groups  **Setting:** hospital | **Time Horizon:** lifetime  **Perspective** not stated  **Decision tree**  **Clinical data:** Published literature and expert opinion.  **Costs:**  Direct costs: screening costs (HBsAg) and costs of selective vaccination (vaccine doses and booster).  Indirect costs: productivity loss due to morbidity and mortality. Discounted at 3%.  **Outcome measures:**  Life-year saved. Discounted at 3% |
