# Supplementary material for: Cost-Effectiveness of HBV and HCV Screening Strategies – A Systematic Review of Existing Modelling Techniques
Source: PLoS One. 2015 Dec 21;10(12):e0145022. doi: 10.1371/journal.pone.0145022 (PMC4686364; doi:10.1371/journal.pone.0145022)
Supplement: S2 Table — (DOCX) [file pone.0145022.s002.docx]

**S4 Table - Evidence Summary HCV**

| **Study Details** | **Population** | **Intervention/Comparator** | **Methods** |
| --- | --- | --- | --- |
| Castelnuovo et al; 2006 [[24](#_ENREF_24)]  UK  £ (price year 2004) | Former IDUs**;** Prison inmates; People in contact with general practice (hypothetical cohort of 1,000)  **Prevalence:**  Male: 54% (36%)  Female: 51% (33%)  All: 53% (35%)  A pooled estimate of 49% (36;61) was used | Testing for HCV infection and offer antibody and if necessary RNA testing  (1) Chronic HCV: 48 week pegylated interferon and ribvarin standard doses  (2)Genotypes 1 and 4 offered biopsy  (3) moderate/ severe cases offered treatment – mild HCV with genotype 1 and 4 monitoring with subsequent treatment if severity advances  Comparator: no screening  **Setting**:  General practice, prison, services for people who misuse drugs and alcohol  Identification of target group in each setting | **Time horizon**: Life time  **Perspective**:  **Decision Tree+ Markov Model**  **Input parameters:** Parameter estimates obtained from literature searches and expert opinion; Progression to cirrhosis estimated from Meta Analysis; Transition probabilities obtained from literature  **Costs:** Obtained from trial of treatment for mild HCV. Discounted at 6%  **Outcome measures:** Cases and deaths prevented;  Life years gained; QALYs gained. Discounted at 1.5% |
| Cipriano et al; 2012  US  USD (Price year 2009)  **Study conclusions:**  Screening for both HIV and HCV is modestly cost-effective (compared to no screening), more frequency screening for HIV provides more benefit at less cost. | 3 risk groups: current IDUs, IDUs in ORT, and non-IDUs  **Prevalence:**  IDUs:  HCV: 35%  HIV: 6.5%  Non-IDUs: HCV: 1.3%  HIV: 0.4% | **Intervention:** Simultaneous screening for HCV and HIV in IDUs who are in opioid replacement therapy (ORT)  **Comparator:** “do nothing”  **Setting:** model to  simulate the population of a representative large U.S. city with 2.5 million persons aged 15 to 59. | **Time horizon:**  Lifetime  **Perspective:** societal  **Dynamic model**  **Input parameters:** published literature, expert opinion  **Cost data:** Medicare  reimbursement rates, modelling study of costs of HIV health states, CDC estimates and Medicare for screening costs.  **Outcome measures:** LYs and QALYs  **Discount rate:**  3% |
| Coffin et al, 2012 [[31](#_ENREF_31)]  US  USD (price year 2010)  **Study conclusions:**  Broader screening for HCV would likely be cost-effective, but would require improved rates of referral, treatment and cure. | General, adult population, aged 20-69 years  **Prevalence:** 0.5% unaware, (varied in SA from 0.49 to 0.75%) | 1. Adding one-time screening of the general adult population to current risk-factor based screening 2. Current standard of care (Risk-factor screening) | **Time Horizon:** Lifetime  **Perspective:** Societal  **Decision Tree plus Markov Model**  **Input parameters:** obtained from the published literature  **Costs:** direct costs for lab costs and office visits, treatment etc. Discounted at 3%  **Outcome measures:** QALYs**;** discounted at 3% |
| Deuffic-Burban et al; 2009 [[53](#_ENREF_53)]  France  Euros (price year 2006)  **Study conclusions:**  In HCWs, early HCV testing (at 1 month after exposure) leads to lower risk of progression, and was shown to be cost-effective. | Healthcare workers: hypothetical cohort of 7,300 HCV-sero-negatives  **Prevalence:**  not specified | (1): **French recommendation**- monitoring of anti-HCV antibodies and ALT activity at 1, 3 and 6 months after exposure and HCV RNA testing to confirm positive anti-HCV antibody results  (2): **European recommendation**- monthly monitoring of ALT activity for 4 months after exposure and of anti-HCV antibodies at 6 months and HCV RNA testing  (3): **Baseline US recommendation**: anti HCV antibody and ALT activity monitoring at 6 month after exposure and HCV RNA to confirm positive anti-HCV antibody results  4): **Alternative US recommendation:** HCV RNA testing 1 month after exposure  **Setting:** Secondary Care | **Time horizon**: lifetime  **Perspective:** societal  **Decision Tree**  **Input parameters:** HCV transmission probability, test sensitivity and specificity, and treatment efficacy (published literature).  **Costs:** screening tests, out-patient visits, treatment of HCV and treatment and follow-up of CHC for non-responders and relapsers ‘French Nomenclature des Actes de Biologie Medicale’ and ‘French Nomenclature Generale des actes professionnels’. Discounted at 3%,  **Outcome measures:** CHC avoided by early therapy; QALYs gained. |
| Eckman et al; 2013 [[34](#_ENREF_34)]  US  USD (Price year 2011)  **Study conclusions:**  Targeted screening is cost-effective when prevalence of HCV exceeds 0.84%. | Ethnic and gender-mixed general population: 49% male, 78% white, 13% African American, and 9% Hispanic, mean age 46 years.  **Prevalence:** 1.4% | Guideline-based screening in populations with varying prevalence of HCV and risks for fibrosis progression  **Comparator:** no screening    **Setting:** Community setting. | **Time horizon:** lifetime  **Perspective:** healthcare system  **Decision tree plus Markov model**  **Input parameters:** NHANES survey for prevalence, published literature for fibrosis progression, American Association for the Study of Liver Diseases guidelines for treatment of chronic HCV.  **Cost data:** published literature, not including indirect costs.  **Outcome measures:** QALYs  **Discount rate:** 3% |
| Honeycutt et al; 2007 [[38](#_ENREF_38)]  US  USD (price year 2006)  **Study conclusions:**  Testing IDUs in STD clinics was associated with the lowest cost per true positive who returned for test results than testing non-IDUs aged ≥40 years. | Individuals attending STD clinics according to subgroups:  (1) IDUs  Non-IDUs aged ≥40 years:  (2) Men ≥40 years with history of ≥100 sexual partners  (3) Men ≥40 years with <100 lifetime sexual partners  (4) Women ≥40 years  **Prevalence (95%CI):**  (1) 57% (44;69)  (2) 16% (6.7;25)  (3) 2% (1.2;2.8)  (4) 0.9% (0.2;1.7) | HCV counselling, testing and referral in four population subgroups.  **Setting:** public STD clinics | **Time horizon:** not reported  **Perspective**: provider  (STD Clinic perspective)  **No model (multiplicative formula)**  **Input parameters:** Effectiveness – estimated percentage of individuals targeted for HCV testing who had a true positive and returned for results – from NHANES (1999-2002) and studies published between 2003 and 2005.  **Costs:** Direct costs to STD clinic providing service (screening, pre-test counselling, risk assessment, blood test, lab analysis and post-test counselling). Discounting not appropriate (costs incurred immediately).  **Outcome measures:** Number of STD clinic patients in each subgroup with true-positive HCV test result, who returned to receive their results. |
| Josset et al; 2004 [[39](#_ENREF_39)]  France  Euros (price year not stated)  **Study conclusions**:  Extension of screening populations beyond risk groups of transfused patients and illicit drug users requires substantial increase in health care costs. | Attendees of general practice, with at least one risk factor, but unknown HCV status:  (1) History of gastroscopy;  (2) contact with infected person;  (3) history of invasive procedures (fluid aspiration, cytology, biopsy);  (4) history of colonoscopy;  (5) history of surgery;  (6) blood transfusion before 1991or date unknown, or present or former IDU or inhaling drug user  Free screening test proposed to patients with at least one of the above risk factors  **Prevalence rate**: 2.6% | Screening test with EIA3, reference strategy = risk group (6)  **Comparator:** Screening tests for remaining risk groups (1) to (5)  **Setting:** Primary care | **Time horizon:** not reported  **Perspective**: not stated  **NO MODEL**  **Input parameters**: Taken from the 1997 survey carried out in GP practices  **Costs:** Only direct medical costs French reimbursement schedules; 3 funding modalities:  (1) screening campaign does not change routine practice and is integral part of routine medical care (T); (2) GPs receive extra fixed payment for screening, irrespective of number of tests – two levels (F); (3) public campaign increases number of consultations and GP activity is affected- three levels (C)  No discounting.  **Outcome measures:** Mean cost per case of HCV infection detected;  MCER between baseline strategy (6) and each of the other 5 strategies |
| Josset et al; 2004  France  Euros (price year 1997)  **Study conclusion:**  Systematic screening before transfusion only was the most cost-effective strategy | Blood transfusion recipients, pre and post transfusion | (1) lack of systematic HCV screening in routine = **PA96,** according to the medical practices observed before 1996 in France.  (2) HCV screening before transfusion and three months after transfusion as recommended since 1996 = **C3**  (3) HCV screening before transfusion and six months after transfusion to limit the number of patients followed up (transfusion is related to poor prognostic)  = **C6**  (4) HCV screening before  transfusion, only, as the residual risk of transfusion-transmitted HCV infection is very low  and the percentages of HCV infection detected before transfusion (=**Cpt)**  **Setting**: Hospital | **Time horizon:**  **Perspective: Health Care system**  **Decision Tree**  **Input parameters:**  **Costs:** direct costs of testing  **Outcome measures:**  Infections detected, MCER |
| Jusot and Colin; 2001 [[48](#_ENREF_48)]  France  French Francs (price year 1996)  **Study conclusions:**  The study showed high costs and few life years gained for the dominant strategy even for the more favourable risk groups | Blood recipients  **Prevalence rate:** 3% (varied from 1 to 9% in SA) | Screening via alanine aminotransferase (ALT), 3^rd^ generation enzyme immuneassay (EIA 3) or detection of HCV RNA in:  (1) young adults <40 years;  (2) blood recipients who had received 1-10 units of red blood cells or were hospitalised in a surgery department or were aged between 40 and 65;  (3) blood recipients, who received >10 units of red blood cells  No testing and no medical therapy was also compared.  **Setting:** Hospital | **Time horizon:** 30 years  **Perspective**: Healthcare System  **Decision Tree+ Markov Model**  **Input parameters:** from literature; Progression rates calculated as annual constant probabilities using published evidence; Annual rate of chronic HCV from literature; Long-term success was estimated from Meta-Analysis; Survival curves calculated using data from the literature  **Costs:** Costs of biological tests (ALT, EIA3 and HCV RNA) and of interferon; Cost of interferon therapy calculated for a) long-term response and b) no response after 12 weeks; Costs also calculated for liver biopsy, follow-up of hep stages and transplantation  **Outcome measures:** LYG |
| Lapane et al; 1998 [[25](#_ENREF_25)]  US  USD (price year not stated)  **Study conclusions:**  Predicted risk based screening – based on risk prediction equation or questionnaires were shown to be more cost-effective than screening according to ALT values. | Retrospective cohort study based on  13,997 self referred individuals screened for viral hepatitis  (National Hepatitis Surveillance Programme, NHSP)  **Prevalence rates:**   1. 20% 2. 29% 3. 25% 4. 12% | 1. Test offered to those who had >7% probability of having HCV, according to a mathematical predictive equation. 2. Test offered to those at significant risk, according to responses to all the questions of a survey. 3. Test offered to those at significant risk, according to responses to all the questions, with the exception of socially intrusive questions, of a survey. 4. ALT testing followed by HCV testing in those with elevated ALT values.   **Setting:** not specified | **Time horizon:** not stated  **Perspective**: not stated  **No model**  **Input parameter:** Effectiveness data were derived from single study (Szklo,1990)  **Costs:** Direct costs included costs for testing (assigned as mean actual charges of those blood tests). No discounting; no future costs were taken into account.  **Outcome Measures:** Number of cases detected; costs per case detected |
| Leal et al; 1999 [[40](#_ENREF_40)]  UK  £ (price year 1997)  **Study conclusions:**  Although potentially cost-effective, major uncertainties remain regarding assumptions made on long-term effectiveness of screening and treatment. | Asymptomatic IDUs (hypothetical cohort)  **Prevalence rate**: 60% | One prevalence round of screening in IDU population  **Comparator:** no screening  **Setting**: not clearly specified – IDUs in contact with services in the South and West health region of the UK | **Time horizon:** 30 years  **Perspective:** not stated  **Decision Tree**  **Input parameters:** Obtained from the published literature: Proportion who accept biopsy, proportion with diseases, acceptance rate for treatment.  **Costs:** Direct costs for screening and diagnosis, counselling, treatment, adverse events, monitoring. Discounted at 6%  **Outcome measures**:  QALY. Only discounted in SA |
| Linas et al; 2012 [[52](#_ENREF_52)]  US  USD (Price year 2011)  **Study conclusions:**  Screening for acute HCV infection in HIV-infected MSM prolongs life expectancy and is cost-  effective. | HIV-infected MSM enrolling in US  Guideline – concordant HIV care.  **Prevalence:** 9.8% | 10 screening strategies:  (1) symptom-based screening,  (2) LFTs every 3 months,  (3) LFTs every 6 months,  (4) LFTs every 12 months, (5) LFTs every 6 months and HCV Ab test every 12 months (NEAT-recommended strategy),  (6) LFTs and HCV Ab test every 3 months,  (7) LFTs and HCV Ab test every 6 months,  (8) LFTs every 6 months and HCV RNA test every 12 months,  (9) LFTs and HCV RNA  test every 3 months,  (10) LFTs and HCV RNA test every 6 months  **Comparator:** symptom-based screening alone    **Setting:** U.S. healthcare system | **Time horizon:** lifetime  **Perspective:** societal  **HEP-CE (HCV cost-effectiveness) model (Monte Carlo model).**  **Input parameters:** cohort characteristics from published reports; HCV incidence from reports of HIV infected MSM; Acute & chronic HCV infection therapy and HCV/HIV disease progression data were obtained through modelling.  **Cost data:** published literature.  **Outcome measures:** LE, QALE, QALY  **Discount rate:**  3% |
| Liu et al; 2013 [[35](#_ENREF_35)]  US  USD (Price year 2010)  **Study conclusions:**  Cost-effectiveness of one-time birth-cohort HCV screening for 40–64 year olds is comparable to other screening programs and likely cost-effective if sufficiently high treatment uptake is ensured. | 40–74 year-old asymptotic U.S. adults who are unaware of their HCV infection status.  **Prevalence:**  11-17% among high-risk individuals  2-3% among low-risk individuals | Screening strategies were assessed in combination with treatment strategies.  1) No screening,  2) Risk-based screening,  3) Birth-cohort screening.  Treatment strategies:  1) Standard therapy,  2) Universal triple therapy,  3) IL-28B-guided triple therapy  **Comparator:** Standard therapy    **Setting:** routine medical visit | **Time horizon:** lifetime  **Perspective:** societal  **Markov model**  **Input parameters:** natural history obtained from published empirically-calibrated model. NHANES survey for risk data. Mortality from NHANES and U.S. life-tables.  **Cost data:** Included out-of-pocket expenses, time lost using hourly wage data, state-specific costs for HCV. All from published sources.  **Outcome measures:** QALYs  **Discount rate:** 3% |
| Loubiere et al; 1999 [[27](#_ENREF_27)]  France  French Francs (price year not reported)  **Study conclusions:**  The strategy of ELISA followed by another ELISA confirmatory test among IDUs was the most cost-effective strategy. | Multiple populations:  IDUs  Recipients of blood transfusions  General Population | Analysis of 5 screening strategies:  1) PCR  2) ELISA  3) ELISA followed by ELISA if tested positive  4)ELISA followed by RIBA if tested positive  5) Two ELISA test in parallel  Following pos. diagnosis, 2 treatment strategies:  (i)Those previously untreated received interferon for 12 months; patients who had relapsed received 6 months of interferon and 6 months of ribavirine  (iI)Those previously untreated received 12 months of interferon and ribavirine; patients who had relapsed received 6 months of interferon and ribavirine.  Status quo of having no screening policy for HCV was also compared.  **Setting:** not specified | **Time horizon:** 10 years  **Perspective**: healthcare system  **Decision Tree + Markov**  **Input parameters:** obtained from published literature  **Costs**: Direct costs of diagnostic testing, consultation, screening, additional testing, treatment. Discounted at 3%  **Outcome measures**: cases avoided |
| Loubiere et al, 2003 [[26](#_ENREF_26)]  France  Euros (price year 1998); also presented in USD  **Study conclusions:**  ‘Wait and treat cirrhosis’ was more cost-effective than either of the screening strategies among the blood recipients and the general population. However, among IDUs screening was more cost-effective than no screening. | Multiple populations:  IDUs  Recipients of blood transfusions  General Population  **Prevalence rates:**   - IDUs: 80% - Blood recipients: 7% - General population: 1.2% | 1. No HCV screening or treatment, but management of complications (cirrhosis etc) 2. ‘Wait and treat cirrhosis’ – initiation of HCV treatment after cirrhosis (symptomatic) 3. Screening via EIA test follows first positive EIA test* 4. Screening via PCR test added to first positive EIA test*   *(3) and (4) refer to current French policy  **Setting**: not specified | **Time horizon**: Lifetime  **Decision Tree + Markov Model**  **Perspective:** Health Care System  **Input Parameters:** Sensitivity and specificity of clinical tests (EIA and PCR) derived from literature  **Costs:** French social insurance tariff; Cost of screening, pre-treatment testing, treating and managing HCV disease;  Costs include inpatient, outpatient, drug costs, additional costs for false positives and false negatives  Excluding non-medical direct costs and indirect costs. Discounted at 3%  **Outcome measures:** Infections detected;  HCV cases avoided;  LYG. Discounted at 3% |
| McGarry et al; 2012 [[32](#_ENREF_32)]  US  USD (price year 2010)  **Study conclusions:**  Birth cohort screening for HCV in previously undiagnosed individuals is likely to be cost-effective at conventional levels of WTP thresholds. | Birth cohort (1946-1970)  **Prevalence:** estimated in preliminary analysis | Targeted birth cohort screening programme of U.S. residents over a total period of 5 years (one-time screening)  **Comparator:** risk based screening as currently undertaken in the US | **Time horizon:** Lifetime  **Perspective:** Payer  **Decision Tree plus Markov Model**  **Input parameters:** obtained from the published literature, other secondary sources and expert opinion  **Costs:** direct costs of HCV diagnosis, management and treatment estimated from claims study and Medicare reimbursement**.** Discounted at 3%  **Outcome measures:**  Deaths averted, QALY; discounted at 3%. |
| Miners et al; 2014 [[51](#_ENREF_51)]  UK  £ (Price year)  **Study conclusions:** the evaluation demonstrates that testing UK migrants for HCV could be cost-effective. | Migrants from the Indian subcontinent, primarily  Bangladesh, India and Pakistan.  **Prevalence:** 3.2% (values of 1% and 5% were used in sensitivity analyses). | Screening (Antibody test for HCV in migrant population)  **Comparator:** no intervention  **Setting:** community primary care setting – patients were invited to clinic via an opt-out strategy | **Time horizon:** lifetime  **Perspective:** NHS  **Markov model.**  **Input parameters:** published literature (2007 HTA report, UK Health Protection Agency data)  **Cost data:** assumptions for carrying out intervention (screening) and published literature for treatment costs.  **Outcome measures:** QALYs  **Discount rate:** 3.5% |
| Nakamura et al; 2008 [[28](#_ENREF_28)]  Japan  USD (price year 2007)  **Study conclusions:**  Screening in both the general population and the high risk group appeared to be more cost-effective than no screening. | General population (N=99,001)  High risk groups (N=42,358), defined as:  - high level of aminotransferase,  - major surgery,  - received blood transfusion during child birth  **Prevalence:**  Genotype 1 - 70%  Genotype 2&3 - 30% | 1. Screening for HCV in general population and high risk groups- every 5 years from age 40 to 70 2. No screening   **Setting**: Outpatient, secondary care | **Time horizon:** 30 years  **Perspective**: not stated  **Published Markov Model (Nakamura et al, 2007)**  **Input parameters:** Transition probabilities, prevalence and effectiveness data of treatment obtained from literature  **Costs:** Only direct costs; overall cost for screening was determined to be the sum of the screening cost for detection and the lifetime HC costs for the patient (based on Markov Model). Costs discounted at 3%  **Outcome measures:** LYG; Discounted at 3% |
| Plunkett et al; 2005 [[49](#_ENREF_49)]  US  USD (price year 2003)  **Study conclusions:**  Screening women during pregnancy for HCV and subsequent treatment for progressive disease were not cost-effective.  The disutility of knowledge of HCV infection for mother and child is not outweighed by the potential benefit of treatment. | Hypothetical cohort of women during pregnancy (30 years)  **Prevalence rate**: 1% (base case), varied from 1 to 10% in SA) | 1) Routine HCV screening in pregnancy and subsequent treatment (48 week course) for progressive disease)  2) HCV screening in pregnancy, with subsequent treatment for progressive disease and elective Caesarean delivery to avert perinatal transmission  3) Usual care (no screening)  **Setting:** Secondary Care | **Time horizon:** Lifetime  **Perspective**: Healthcare system  **Decision Tree + Markov model**  **Input parameters**: Epidemiological parameters relating to disease progression, probability of different modes of delivery and probability of perinatal transmission (from published literature).  **Costs**: Direct costs only (pre- and post-test counselling, screening tests, delivery procedures, annual costs associated with the management of patients). Discounted at 3%  **Outcome measures**:  QALYs for mother and child. Discounted at 3%. |
| Rein et al; 2012 [[33](#_ENREF_33)]  US  USD (price year 2010)  **Study conclusions:**  Birth-cohort screening seems to be a reasonable strategy to identify asymptomatic cases of HCV. | Birth cohort (1945-1965) with one or more visits to a primary care provider in 2006  **Prevalence:** no assumptions stated | 1. No screening or treatment 2. Risk based screening 3. Birth-cohort screening (one-time) then offering standard treatment 4. Identical birth-cohort screening scenario with genotype 1 patients receiving additional treatment   Setting: primary care | **Time horizon:** lifetime  **Perspective:** societal, health care  **Markov Model**  **Input parameters:** obtained from National Health and Nutrition Examination Survey, U.S. Census, Medicare reimbursement schedule, and published sources.  **Costs:** medical and productivity costs. Discounted at 3%.  **Outcome measures:**  cases identified, treated and achieved a sustained viral response; liver disease and death from HCV; QALYs; discounted at 3% |
| Ruggeri et al; 2013 [[36](#_ENREF_36)]  Italy  Euros (Price year 2009)  **Study conclusions:** An anti-HCV screening program is a valid health-related investment improving patients’ quality of life and survival with an acceptable expenditure increase for the health service. Results are driven by patient age at screening. | Hypothetical general population cohort (100,000)  **Prevalence by age group:**  15-30: 2%,  31-45: 6%  46-60: 7%  >60: 5% | **Intervention:** Screening (first level = enzyme immunoassay for HCV antibodies and if positive, second test = HCV RNA) and treatment of subjects testing positive  **Comparator:** no-screening and treatment only of patients with cirrhosis or HCC  **Setting:** Italian health care system | **Time Horizon:** lifetime  **Perspective**: health care service  **Markov model**  **Input parameters:** published literature  **Cost data:** Italian Agency of Medicine, Official Gazette no. 289 of December 13, 2006, Diagnosis Related Group, and expert opinion.  **Outcome measures:** QALYs  **Discount rate:** 3.5% |
| Schackman et al; 2014 [[47](#_ENREF_47)]  US  USD (Price year 2011)  **Study conclusions:**  On-site rapid hepatitis C virus and HIV testing in substance abuse treatment programs is cost-effective at a <$100, 000/quality-adjusted life-year threshold | IDUs who do not report being HCV- or HIV-  infected in substance abuse treatment centres  **Prevalence:**  HCV: 11%  HIV: 0.4% | **Intervention:**  (i) no HCV test referral or offer (no intervention);  (ii) referral to an off-site HCV antibody test;  (iii) offer of an on-site rapid HCV antibody test;  (iv) offer of on-site rapid HCV and HIV antibody tests.  Tests were combined with three different treatment scenarios:  (i) Interferon  (ii) SOF based regimes  (iii) interferon-free regimes  **Comparator:** do nothing  **Setting:** Substance abuse treatment centres | **Time Horizon:** Lifetime  **Perspective**: healthcare system  **Decision tree plus HEP-CE or CEPAC model**  **Input parameters:** published literature  **Cost data:** published literature  **Outcome measures:** QALYs  **Discount rate:** 3% |
| Singer et al; 2001 [[29](#_ENREF_29)]  US  USD (price year 2001)  **Study conclusions:**  Screening of average risk adults in the general population in primary care was shown to be not cost-effective, when compared with no screening. | Average risk adult population presenting to their primary healthcare provider without any specific symptoms  **Prevalence:**  2.9% (varied from 0.8 to 9% in SA) | 1. Initial screening with ELISA, confirmation with PCR 2. PCR: initial screening by PCR 3. No screening (current practice)   **Setting**: Primary Care | **Time horizon**: lifetime  **Perspective**: Societal  **Decision Tree+ Markov Model ((cohort and Monte Carlo simulations)**  **Input parameters:** obtained from the literature; Transition rates to cirrhosis, transplantation and liver cancer.  **Costs**: Direct and indirect costs: screening tests, genotyping, examinations, treatment and management care plus costs due to loss of work. Discounted at 3%.  **Outcome measures:** QALYs; discounted at 3%. |
| Stein et al; 2003 [[30](#_ENREF_30)]  UK  £ (price year 2001)  **Study conclusions:**  Universal screening is unlikely to be cost-effective. | Hypothetical cohort of individuals attending GUM clinics (clinical risk factors)  **Prevalence:** 1.5% (base case) | (1) Universal screening of attendees of GUM clinics  (2) Restricted screening (current or former IDUs)  (3) Screening in larger minority of attendees, thus allowing flexibility of defining ‘at risk’ population  **Comparator**: No screening  **Setting:** GUM clinics | **Time horizon:** 50 years  **Perspective**: NHS  **Decision Tree + Markov Model**  **Input parameters**: Obtained from the published literature (good quality studies, where possible) and clinician /expert opinion  **Costs:** Direct costs for: assessing eligibility, counselling, ELISA and PCR tests, liver biopsy, GP visit, outpatient visit and inpatient stay, drug costs, treatment costs. Discounted at 6%.  **Outcome measures:**  QALYs; discounted at 1.5%. |
| Stein et al, 2004 [[41](#_ENREF_41)]  UK  £ (price year 2001)  **Study conclusions:**  Screening for HCV in IDUs in contact with services is moderately cost-effective and reasonably stable when explored in one-way SA. | Hypothetical population of individuals with history of injecting drugs, who are currently in contact with misuse services | Screening and treating IDUs in one prevalent round of screening  **Comparator**: No Screening  **Setting:** Primary care | **Time Horizon**: 50 years  **Perspective:** NHS  **Decision Tree + Markov Model**  **Input parameters:** obtained from a review of the literature and expert opinion  **Costs**: Direct costs for: assessing eligibility, counselling, ELISA and PCR tests, liver biopsy, treatment. Discounted at 6%  **Outcome measures:** QALYs, Costs per QALY. Discounted at 1.5% |
| Sutton et al; 2006 [[42](#_ENREF_42)]  UK  £ (price year 2004)  **Study conclusions:**  Verbal screening for those previously having received a positive HCV test and for ever having injected illicit drugs was the most cost-effective option and had the smallest budgetary impact. | Prisoners (on entry into prison) in the Isle of Wight prison cluster  **(targeting IDUs)** | 1. Verbally screen for ever having received positive HCV test in the past and for ever having injected illicit drugs 2. Verbally screen for past positive HCV test only 3. Verbally screen for injecting illicit drugs only 4. No verbal screening 5. Do nothing (no screening and no testing)   **Setting: Prison** | **Time horizon:** 2006-2017  **Perspective**: Healthcare provider  **Markov Model**  **Input parameter**: review and synthesis of published studies  **Costs:** Direct costs: cost of time taken for all aspects of the verbal tests, ELISA and PCR tests, counselling + unit costs for doctor and nurse time. Discounted at 3.5%  **Outcome measures:** cases detected; discounted at 3.5%. |
| Sutton et al; 2008 [[43](#_ENREF_43)]  UK  £ (price year 2004)  **Study conclusions:**  Results at baseline would suggest that screening and treatment for HCV in prisons is not cost-effective. The results are subject to much uncertainty. | Prisoners at different ages | Case finding arm: Testing and treatment of people entering prison with the possibility of later spontaneous presentation for screening and treatment in a community location  **Comparator:** presentation for screening and treatment only possible in a community location  **Setting**: Prison and in the community. | **Time horizon**: 80 years  **Perspective**: Healthcare Provider  **Markov Model**  **Input parameters:** obtained from the literature  **Costs:** costs for test, clinical consultation, genotyping and treatment associated with disease states, derived from the published literature. Discounted at 3.5%.  **Outcome measures:** QALYS; discounted at 3.5%. |
| Thompson Coon et al; 2006 [[44](#_ENREF_44)]  UK  £ (price year 2004)  **Study conclusions:**  Case finding for HCV in primary care is likely to be cost-effective but substantial uncertainties remain. | Former IDUs  **Prevalence:**  12.5% (used in the population strategy)  49% (used in the population targeted strategy) | 1. Offer of testing to all those within a target age group (population approach); 2. Offering testing to those known to be at the highest risk of having contracted HCV (targeted approach)   **Comparator**: A non-case finding approach where individuals were permitted to present spontaneously for testing.  **Setting**: Primary Care | **Time horizon:** Lifetime  **Perspective**: NHS  **Decision Tree + Markov**  **Input parameters:** obtained from the published literature  **Costs:** Direct costs of patient identification and contact; consultation time; counselling and testing; costs for treatment. Discounted at 6%.  **Outcome measures:**  QALYs; discounted at 1.5%. |
| Tramarin et al; 2008 [[45](#_ENREF_45)]  Italy  Euros (price year not reported)  **Study conclusions:**  In the IDU cohort, the screening strategy can result in a substantial difference in premature deaths and dominates the no screening strategy. The number of deaths prevented in the IWSs cohort is lower resulting in greater incremental costs per QALY gained. | - IDUs - Individuals with Surgery   Two cohorts of individuals treated for HCV: CHC (chronic scenario) and AHC (acute or screening scenario) | Screening compared with no screening  Comparison between two strategies (cohorts) is made by following the incident over time within both scenarios, CHC and AHC. CHC scenario was stratified by genotypes. AHC scenario consists of two sub-cohorts exposed to relatively high risks of infection (IDUs and IWSs).  **Setting:** Veneto Region of Italy | **Time horizon:** lifetime  **Perspective:** societal  **Markov Model (mathematical model)**  **Input parameters:** Obtained from literature and published clinical trial results: HCV symptomatic, HCV asymptomatic, proportion of patients with spontaneous clearance of the virus, proportion of patients evolving to a disease. Proportion of patients with AHC and SVR after therapy. Proportion of patients with CHC and SVR after therapy.  **Costs**: no indirect costs, costs derived from Italian National Tariffs System (outpatient visits, lab tests and diagnostic interventions not included) Costs of screening, therapy and having the disease. Discounted at 3%  **Outcome measures:**  QALYs; discounted at 3%. |
| Urbanus et al; 2013 [[50](#_ENREF_50)]  The Netherlands  Euros (Price year 2011)  **Study conclusions:**  Adding HVC screening to existing program for prenant woman is not C-E in general, however, for 1st generation non-Western woman a modest C-E outome is shown. This is only the case for standard treatment but not for adding protease inhibitors to standard treatment. | All pregnant women and  first-generation pregnant migrants from non-Western countries  **Prevalence:**  All women: 0.2%  non-Western women: 0.43%. | Scenario 1a and b: no screening of either all pregnant or non-Western pregnant women (current practice)  Scenario 2a: screening all pregnant women plus standard treatment  Scenario 2b: screening only non-Western pregnant women plus standard treatment  Scenario 3a and b: as Scenario 2, but with addition of protease inhibitors to standard treatment for all genotypes  **Comparator:** no screening  **Setting:** adding HCV screening to routine screening for pregnant women in primary care. | **Time horizon:** lifetime  **Perspective:** health care system  **Markov model**  **Input parameters:** 2003 routine screening for pregnant women in Amsterdam area – retrospectively screened for HCV  **Cost data:** published literature  **Outcome measures:** Life-years gained  **Discount rate:** costs: 4%, life-years: 1.5%. |
| Wong et al; 2015 [[37](#_ENREF_37)]  Canada  Canadian $ (Price year 2012)  **Study conclusions:** A selective one-time HCV screening program for people 25–64 or 45–64 years of age in Canada would likely be cost-effective. | Canadian residents (general population) in  two age groups:  25–64 years and 45–64 years.  **Prevalence:**  Age 25-34 and 35-44: 0.4%  Age 45-54 and 55-64: 0.8% | 4 screening strategies:  1) no screening  2) screen and treat with pegylated interferon plus ribavarin  3) screen and treat with pegylated interferon and ribavarin–based DAAs  4) screen and treat with interferon-free DAAs.  **Comparator:** no screening  **Setting:** Canadian health care system | **Time horizon:** Lifetime  **Perspective:** Payer’s perspective  **Markov Model**  **Input parameters:** clinical and utilities estimates from published literature.  **Cost data:** Published literature  **Outcome measures:** QALYs  **Discount rate:** costs and health benefits at 5%. |
|  |  |  |  |
