# Supplementary material for: Cost-Effectiveness of HBV and HCV Screening Strategies – A Systematic Review of Existing Modelling Techniques
Source: PLoS One. 2015 Dec 21;10(12):e0145022. doi: 10.1371/journal.pone.0145022 (PMC4686364; doi:10.1371/journal.pone.0145022)
Supplement: S1 Text — (DOCX) [file pone.0145022.s004.docx]

**S1 Text – Search Strategies**

| **MEDLINE (Ovid) (monthly search) – HCV** | **MEDLINE (Ovid) (monthly search) - HBV** |
| --- | --- |
| 1. economics/ 2. exp "costs and cost analysis"/ 3. economics, dental/ 4. exp "economics, hospital"/ 5. economics, medical/ 6. economics, nursing/ 7. economics, pharmaceutical/ 8. (economic$ or cost or costs or costly or costing or price or prices or pricing or pharmacoeconomic$).ti,ab. 9. (expenditure$ not energy).ti,ab. 10. value for money.ti,ab. 11. budget$.ti,ab. 12. or/1-11 13. ((energy or oxygen) adj cost).ti,ab. 14. (metabolic adj cost).ti,ab. 15. ((energy or oxygen) adj expenditure).ti,ab. 16. or/13-15 17. 12 not 16 18. letter.pt. 19. editorial.pt. 20. historical article.pt. 21. or/18-20 22. 17 not 21 23. Animals/ 24. Humans/ 25. 23 not (23 and 24) 26. 22 not 25 27. hepatitis, viral, human/ 28. exp hepatitis c/ 29. ((hepatitis or hep) adj c).ti,ab. 30. HCV.ti,ab. 31. 27 or 28 or 29 or 30 32. exp mass screening/ 33. test$.mp. 34. case find$.mp. 35. screen$.mp. 36. 32 or 33 or 34 or 35 37. 26 and 31 and 36 | 1. economics/ 2. exp "costs and cost analysis"/ 3. economics, dental/ 4. exp "economics, hospital"/ 5. economics, medical/ 6. economics, nursing/ 7. economics, pharmaceutical/ 8. (economic$ or cost or costs or costly or costing or price or prices or pricing or pharmacoeconomic$).ti,ab. 9. (expenditure$ not energy).ti,ab. 10. value for money.ti,ab. 11. budget$.ti,ab. 12. or/1-11 13. ((energy or oxygen) adj cost).ti,ab. 14. (metabolic adj cost).ti,ab. 15. ((energy or oxygen) adj expenditure).ti,ab. 16. or/13-15 17. 12 not 16 18. letter.pt. 19. editorial.pt. 20. historical article.pt. 21. or/18-20 22. 17 not 21 23. Animals/ 24. Humans/ 25. 23 not (23 and 24) 26. 22 not 25 27. hepatitis, viral, human/ 28. exp hepatitis b/ 29. ((hepatitis or hep) adj b).ti,ab. 30. HBV.ti,ab. 31. 27 or 28 or 29 or 30 32. exp mass screening/ 33. test$.mp. 34. case find$.mp. 35. screen$.mp. 36. 32 or 33 or 34 or 35 37. 26 and 31 and 36 |
