# Supplementary material for: Cost-Effectiveness of HBV and HCV Screening Strategies – A Systematic Review of Existing Modelling Techniques
Source: PLoS One. 2015 Dec 21;10(12):e0145022. doi: 10.1371/journal.pone.0145022 (PMC4686364; doi:10.1371/journal.pone.0145022)
Supplement: S2 Text — (DOCX) [file pone.0145022.s005.docx]

**S2 Text - Methods for Model Critique (Categories of Assessment based on the Philips Checklist)**

| - Study Basic (S1 - S2) | - Data Identification (D1) |
| --- | --- |
| - Structure (S3 - S4) | - Data Modelling (D2a) |
| - Intervention and Comparator (S5) | - Treatment Effects (D2b) |
| - Model Basics (S6 - S7) | - Costs & QALYs (D2c - D2d) |
| - Pathways (S8 - S9) | - Data Incorporation (D3) |
| - Consistency (C1 - C2) | - Uncertainty (D4a – D4d) |

*The coding in brackets refer to the individual questions from the Philips Checklist*

The performance matrix was based on and presented as a ‘traffic light’ system. Each of the individual questions in the Philips checklist were each assigned a colour (red, amber, green) according to their relative importance in an assessment of the quality of an economic model. Questions on criteria that were considered critical to the assessment of the model quality are coloured “red”, questions that were important but not critical were coloured “amber” and items that were judged not to alter any conclusions drawn from the analysis were coloured “green”. Possible answers that could be given when assessing the study against the Philips checklists were ‘yes’, ‘no’ or ‘not applicable’. The colour that each individual question got assigned was determined by a negative response (‘no’) to the question. For instance, if questions, regardless of the colour they were assigned, were answered with ‘yes’ they were coloured ‘green’. However, if a question that was assigned the colours ‘red’ or ‘amber’ was answered with ‘no’, this question was coloured ‘red’ or ‘amber’. If a question that was coloured ‘green’ was answered with ‘no’ it stayed ‘green’. This reflects the importance of individual questions as outlined above, i.e. failing on a question that is unlikely to alter conclusions drawn from the model (green) did not affect conclusions in terms of the quality of the economic model.

Individual questions and their assigned colour were subsequently summarised into twelve categories as outlined above (Text Box 1). A category will be coloured amber/red when any negative response occurs to a question assigned amber/red within that category. As such, a negative answer to any of the questions will affect the performance for the entire category if the question was rated important. A category will be coloured green either if all the responses to individual questions within that category are positive (yes) or ‘N/A’ or if all negative responses (no) are to questions that are unlikely to alter conclusions (green).
